# Multiple factors affecting *Ixodes ricinus* ticks and associated pathogens in European temperate ecosystems (northeastern France)

**DOI:** 10.1038/s41598-024-59867-x

**Published:** 2024-04-24

**Authors:** Nathalie Boulanger, Delphine Aran, Armand Maul, Baba Issa Camara, Cathy Barthel, Marie Zaffino, Marie-Claire Lett, Annick Schnitzler, Pascale Bauda

**Affiliations:** 1 Université de Strasbourg UR3073: PHAVI: Groupe Borrelia, 67000 Strasbourg, France; 2 Centre National de Référence Borrelia, Centre Hospitalier Régional Universitaire, Strasbourg, France; 3 Université de Lorraine, CNRS, LIEC, 57000 Metz, France; 4 Université de Strasbourg, MUFJ, 67000 Strasbourg, France; 5 Museum National d’Histoire Naturelle, UMR 7194 HNHP CNRS/MNHN/UPVD, 75000 Paris, France; 6 Université de Lorraine, LCOMS EA 7306, 57073 Metz, France

**Keywords:** Ecology, Biological techniques

## Abstract

In Europe, the main vector of tick-borne zoonoses is *Ixodes ricinus*, which has three life stages. During their development cycle, ticks take three separate blood meals from a wide variety of vertebrate hosts, during which they can acquire and transmit human pathogens such as *Borrelia burgdorferi* sensu lato, the causative agent of Lyme borreliosis. In this study conducted in Northeastern France, we studied the importance of soil type, land use, forest stand type, and temporal dynamics on the abundance of ticks and their associated pathogens. Negative binomial regression modeling of the results indicated that limestone-based soils were more favorable to ticks than sandstone-based soils. The highest tick abundance was observed in forests, particularly among coniferous and mixed stands. We identified an effect of habitat time dynamics in forests and in wetlands: recent forests and current wetlands supported more ticks than stable forests and former wetlands, respectively. We observed a close association between tick abundance and the abundance of Cervidae, Leporidae, and birds. The tick-borne pathogens responsible for Lyme borreliosis, anaplasmosis, and hard tick relapsing fever showed specific habitat preferences and associations with specific animal families. Machine learning algorithms identified soil related variables as the best predictors of tick and pathogen abundance.

## Introduction

Ticks and tick-borne diseases have become a major public health problem due to the expansion of their geographic range and increasing incidence in humans^1,2^. Indeed, the epidemiology of tick-borne disease is constantly evolving^3–5^; some tick-borne diseases are therefore considered emerging diseases^2,6^.

Tick-borne diseases are zoonoses, although humans are accidental hosts. Ecosystem alteration and climate change both favor the proliferation of ticks^7–9^; for example, climate change^10,11^ affects the geographic distribution and density of tick populations, as well as the tick life cycle and the reproductive success of their hosts, in ways that are often tick and host-species specific and therefore difficult to predict^5,12^.

In Europe, the most common tick species is *Ixodes ricinus*, which can transmit several human pathogens including *Borrelia burgdorferi* sensu lato (causative agent of Lyme borreliosis), *Anaplasma phagocytophilum* (causative agent of human anaplasmosis), and *Borrelia miyamotoi*, causative agent of hard tick relapsing fever^13^ (HTRF)*. I. ricinus* is a three-host tick: each life stage (larva, nymph, and female adult) feeds on a different host. Documented hosts for *I. ricinus* include more than 300 vertebrate species^14^, most of which are small- to medium-size mammals (e.g., rodents, hedgehogs, foxes, hares) reptiles and birds^15^. Given the large blood meal taken by female ticks, the host for these stages is represented by large or intermediate-sized mammals, deer being the most important one in most habitats^16^. Bacterial pathogens are mainly transmitted by nymphs and adult females, though nymphs are considered to pose the greater threat to humans in Europe due to its small size and its abundance in the environment^17^. The *Ixodes* tick is mainly found in deciduous or mixed forests which offer leaf litter with sufficient humidity^15^. It is also increasingly found in urbanized areas, particularly in urban green spaces^18–20^.

Medlock et al.^21^ identified three categories of environmental change that are driving the ongoing range expansion of *I. ricinus* in Europe: (1) climate change, which contributes to the geographical expansion into higher latitudes (e.g., Scandinavia) and altitudes (e.g., in central Europe); (2) factors linked to changes in the distribution of tick hosts, such as ecological changes or habitat connectivity; and (3) anthropogenic changes, such as changes in forest management practices, tourism or recreational activities. These factors are not independent of each other, but they are rarely considered simultaneously and can be difficult to quantify. Moreover, to holistically assess the risk *I. ricinus* poses to human health, studies must focus not only the tick but also on the distribution and incidence of microorganisms (bacteria, viruses, parasites) that it can transmit^13,22^. In the present study, we focused on bacteria transmitted by *I. ricinus* since they represent a major health concern in this region^23^. In northeastern France, where tick-borne diseases are endemic, Goldstein et al.^24^ recently showed that the abundance of *Ixodes* nymphs depended on the type of humus, soil relative humidity, and soil composition.

*Ixodes ricinus* is the most abundant tick in Europe and is a particularly important vector for the *B. burgdorferi* sensu lato (s.l.) complex^25^. The prevalence of *B. burgdorferi* s.l. among *I. ricinus* ticks varies with the local characteristics of biocenoses and with the season^26–28^. Climate and local biodiversity are also important drivers of pathogen infection rates, but they are not sufficient to explain all the observed variation in infection rates. Indeed, many studies have called for more detailed analyzes of tick microhabitats^4,21,29^.

Five genospecies of pathogenic *B. burgdorferi* s.l. are common in Europe, though the circulation of each species depends on the dynamics of their respective vertebrate hosts^30^. For example, small mammals are the main reservoir of *B. afzelii*, whereas birds are the main reservoir of *B. garinii* and *B. valaisiana*. The other two genospecies in Europe are *B. burgdorferi* s.s. and *B. spielmanii*^30^. Deer are not competent hosts for any of the five genospecies but are essential for supporting tick populations because of their role in feeding adult females^15^.

In summary, the environmental factors driving local variation of *I. ricinus* abundance and the incidence of Lyme borreliosis in humans are known. These factors are multiple. Humidity is the most important one as well as temperatures for tick activity. Ticks also need different suitable hosts to complete their developmental cycle such as ungulates essential for the successful feeding of adult ticks and, suitable habitat usually mixed forests^16^. Their respective contribution and interaction are not sufficiently studied and still deserve to be further investigated. These abiotic and biotic factors are affected by anthropogenic activities such as forest and hunting practices. Addressing these questions requires the holistic “OneHealth” approach that considers both human societies and their local environment^31^. In other words, an experimental approach that considers soil, plant, and animal biodiversity, as well as human practices and land uses, is essential for understanding the dynamics of ticks and tick-borne diseases^1,32,33^ and for their prevention^34^.

In this study, we address four questions regarding the factors controlling the abundance of ticks and their level of infection in northeastern France. In 2021, Lyme borreliosis incidence in France was 71 cases per 100,000 inhabitants, > 100 in North East of France and > 200 in Alsace, the most endemic region of France^35,36^. Although we focused on Lyme borreliosis due to the prevalence of the *B. burgdorferi* complex, we also considered the *B. miyamotoi* form of relapsing fever (HTRF) and anaplasmosis (caused by *Anaplasma phagocytophilum*)^13^ in spring which corresponds to the peak activity levels of *I. ricinus* in Europe^37,38^. Using an experimental design for the selection of tick collection sites, we explored how the abundance of ticks and tick-borne pathogens are influenced by (1) the mineral substrate and associated soil; (2) land use (orchard, meadow, wetland, or forest), (3) global changes (drying of wetlands to form new meadows and recent forest colonization consecutive to agricultural or economic decline) and (4) the nature of the forest stands (coniferous, deciduous or mixed forest).

## Results

**Figure 1:**
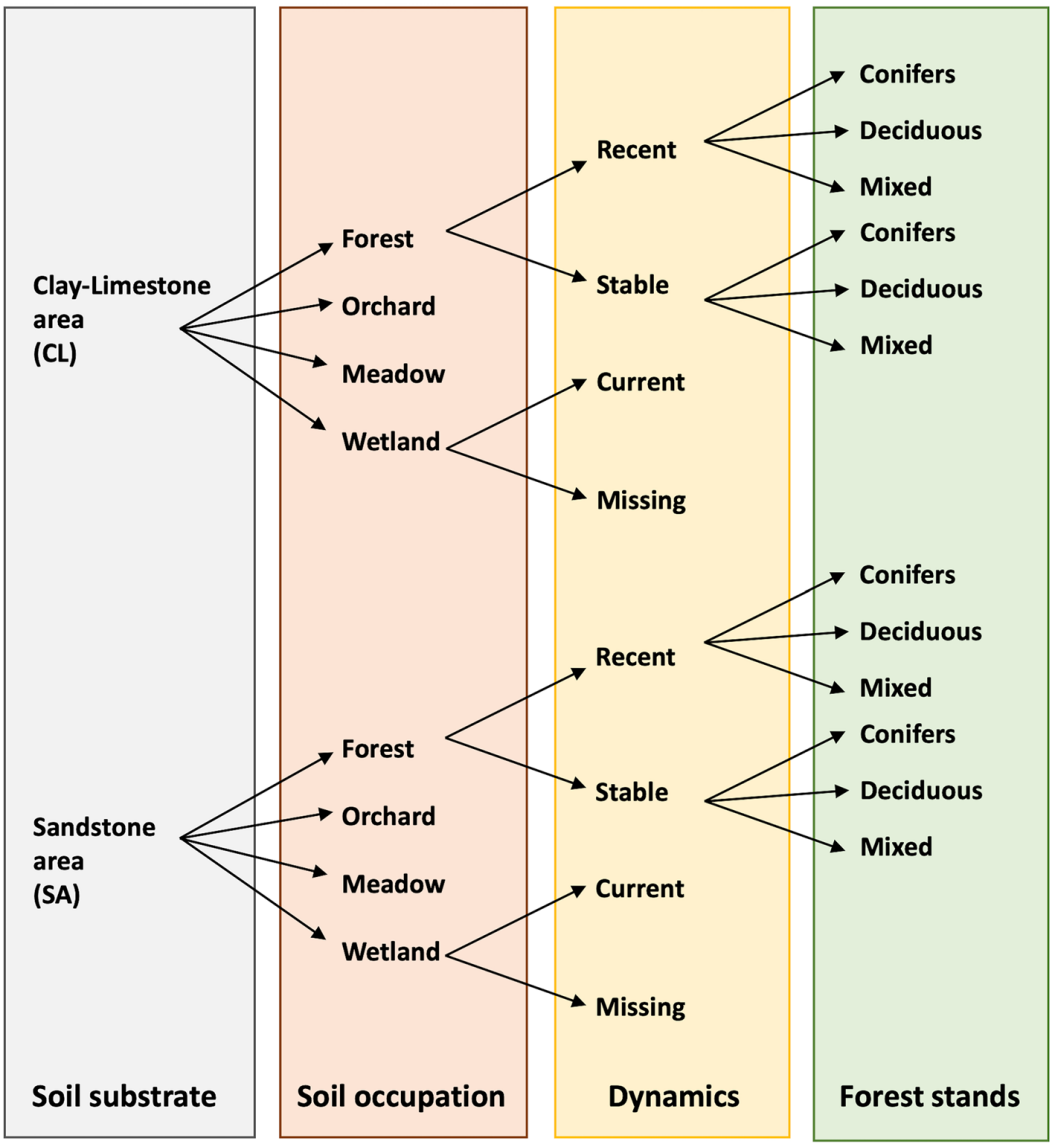
Experimental design for tick collection.

To identify the environmental factors that determine the density of *I. ricinus* and tick-borne pathogens, we carried out four tick surveys in June 2020, April 2021, May 2021, and June 2021. Surveys were conducted at 40 selected sites across two different geological substrates, clay-limestone (20 sites) and sandstone (20 sites), as shown in Fig. 1. Sites were further classified based on their habitat type (forest, orchard, meadow, or wetland), the temporal nature of that habitat (recent/stable for forests, current/missing for wetlands), and forest type (deciduous, coniferous, or mixed, for forested habitat types). Their characteristics are specified in Table 1 and more detailed in Supplementary Material S1.

**Table 1:** Code and signification of the 40-tick sampling sites.

Soil occupation	Soil substrate			
	Clay limestone area		Sandstone area	
	Parcel number	Code	Parcel number	Code
Orchard	1	CL-O	21	SA-O
	2		22	
Meadow	3	CL-Me	23	SA-Me
	4		24	
Wetland current	5	CL-W-C	25	SA-W-C
	6		26	
Wetland missing	7	CL-W-Mis	27	SA-W-Mis
	8		28	
Forest recent conifers	9	CL-F-R-Co	29	SA-F-R-Co
	10		30	
Forest stable conifers	11	CL-F-S-Co	31	SA-F-S-Co
	12		32	
Forest recent deciduous	13	CL-F-R-D	33	SA-F-R-D
	14		34	
Forest stable deciduous	15	CL-F-S-D	35	SA-F-S-D
	16		36	
Forest recent mixed	17	CL-F-R-Mix	37	SA-F-R-Mix
	18		38	
Forest stable mixed	19	CL-F-S-Mix	39	SA-F-S-Mix
	20		40	

### Nymph abundance/seasonal effect

The mean (median) nymph abundance (measured as nymphs/200 m^2^) across all study sites was 19.0 (8.5), 55.3 (9.5), 47.8 (14.5), and 25.5 (4.0) for each of surveys 1, 2, 3, and 4, respectively. Nymph abundance differed significantly across the four surveys (Friedman test with blocks on the 40 parcels examined, T_FR_ = 11.4; p = 0.0098). The analysis indicates a decrease in nymph abundance from April to June in both years we sampled. Furthermore, a pairwise rank correlation analysis between the four surveys showed that nymph abundance was relatively consistent and reproducible between sites across the surveys (Kendall's τ > 0.70; p < 10^–4^). This spatial reproducibility of tick abundance patterns over time allowed us to model nymph abundance as a function of the different environmental variables we considered.

### Nymph abundance/soil substrate/habitats

**Figure 2:**
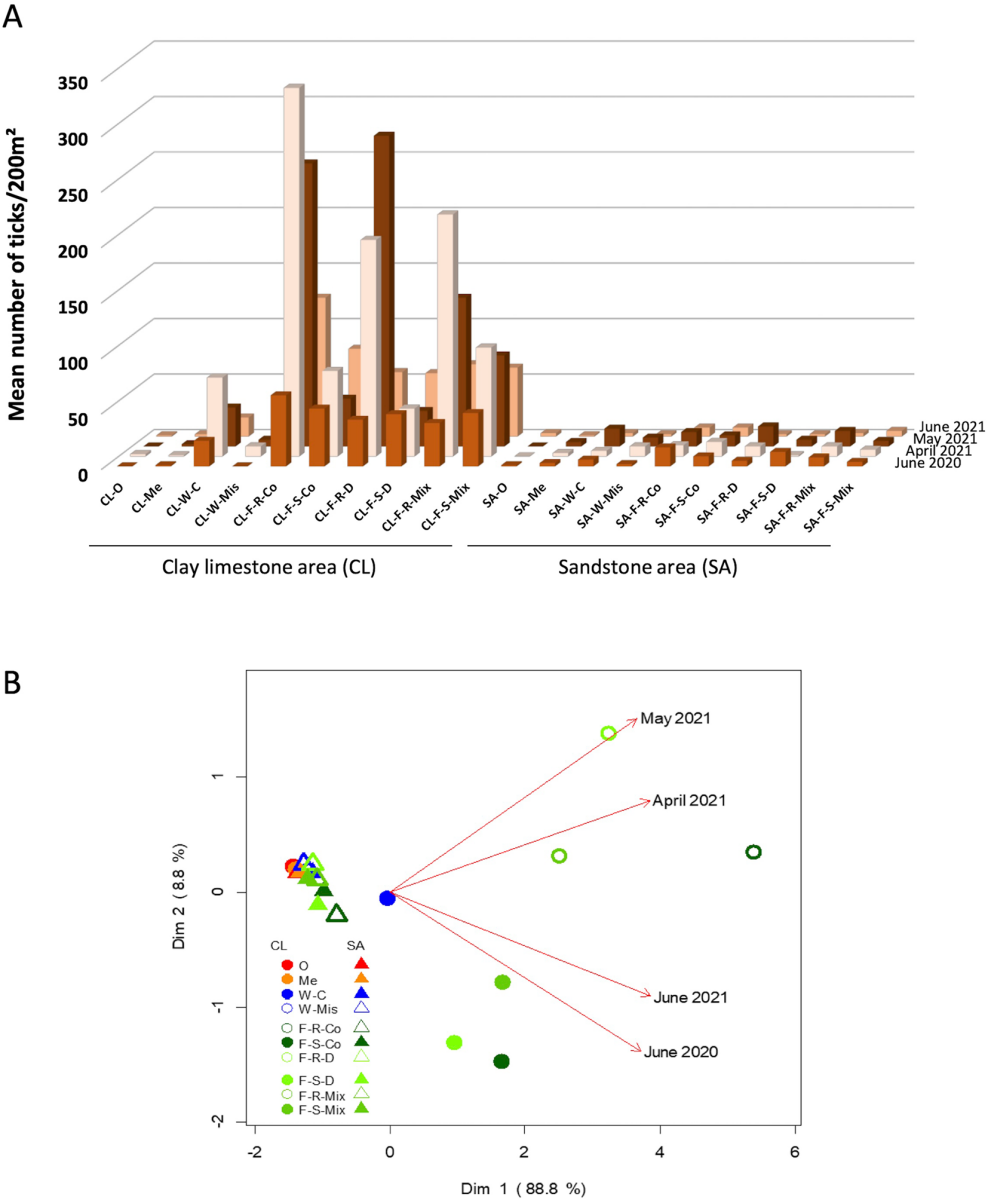
(**A**) Abundance of tick nymphs (mean values of duplicate samples/200 m^2^) shown for each habitat type in each of the four sampling surveys. (**B**) PCA of nymph abundance clustered by sampling sites and surveys. *CL* clay-limestone area, *SA* sandstone area, *O* orchard, *Me* meadow, *W* wetland, *C* current, *Mis* missing, *F* forest, *R* recent, *S* stable, *Co* conifers, *D* deciduous, *Mix* mixed. Code for sampling sites are given in Table 1.

Figure 2A shows the average nymph abundance in each habitat type we tested. Overall, ticks were more abundant on clay-limestone soils than on sandstone soils, where the mean (median) nymph abundance (measured as nymphs/200 m^2^) was (67.5 (39.5)) and (6.3 (5.0)) respectively. Within each soil type, the most favorable habitats for ticks were current wetlands (21.6 (13.5)) and forests (56.5 (17.5)), especially recent forests (79.8 (20.0)). We used PCA to further investigate tick abundance among the 10 different habitat types on both soil types for each of the four surveys. These results are shown in Fig. 2B. As shown by the arrows associated with the four sampling surveys, component 1 of the PCA plot, which explained 88.8% of the total variation in the data, was strongly associated with nymph abundance (r = 0.94). Component 2, which explained 8.8% of the variability, differentiated recent forests (top of Fig. 2B) from stable forests (bottom of Fig. 2B).

### Nymphs and infected nymph abundance versus time and soil substrates

**Figure 3:**
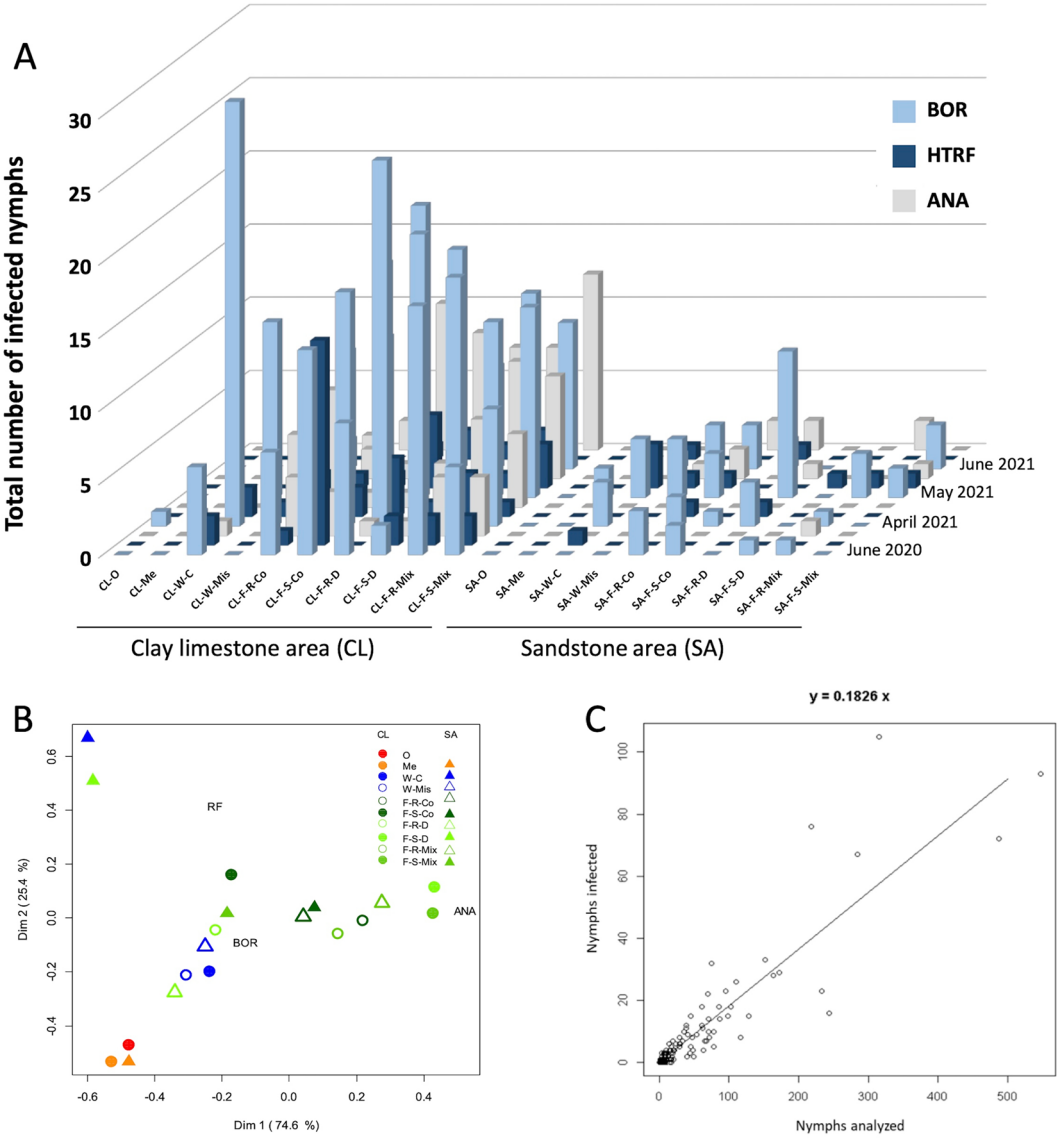
(**A**) Abundance of *Ixodes ricinus* nymphs infected by BOR, HTRF and ANA, collected during the four different surveys. (**B**) Correspondence analysis showing associations between pathogen abundance and soil geophysical characteristics (i.e., soil occupation). (**C**) Regression between the number of nymphs infected and the number of nymphs analyzed. BOR, *Borrelia burgdorferi* sensu lato; HTRF, *Borrelia miyamotoi;* ANA, *Anaplasma phagocytophilum*; *CL* clay-limestone area; *SA* sandstone area; *O* orchard; *Me* meadow; *W* wetland; *C* current; *Mis* missing; *F* forest; *R* recent; *S* stable; *Co* conifers; *D* deciduous; *Mix* mixed. Code for sampling sites are given in Table 1.

The proportions of nymphs infected with *B. burgdorferi* s.l. (BOR), *B. miyamotoi* (HTRF) and *A. phagocytophilum* (ANA) are presented in Fig. 3. Interestingly, most pathogens were more abundant among ticks in clay-limestone sites than in sandstone sites (Fig. 3A).

Based on the results shown in Fig. 3A and supporting statistical analysis, we can conclude that the relative abundance of the three pathogens we considered were not the same across the four sampling surveys (chi-square test; p < 10^–4^). Specifically, HTRF and ANA were under and over-represented, respectively, in survey S4. However, the overall proportion of infected nymphs seemed stable across the four surveys (chi-square test; p = 0.1901), though the abundance of the three pathogens varied according to soil occupation as defined in Fig. 1 (chi-square test; p = 0.0051).

Variation in pathogen abundance due to soil geophysical characteristics is illustrated in more detail in the correspondence analysis (CA) shown in Fig. 3B. Habitat-dependent differences were most apparent for ANA, which appeared to be overrepresented in CL-forests relative to orchard-meadow-wetland environments. Conversely, BOR was overrepresented in CL-orchard-meadow-wetland environments. However, correlations between pathogen abundance and land occupation were also more significant in CL soils because more nymphs were collected in those habitats; the number of analyzed nymphs in SA soils was too low to produce meaningful correlations.

The results in Table 2 show that, during the four sampling surveys, the mean proportions of nymphs with BOR, HTRF, and ANA were 12.2% (9.4–14.3%), 2.2% (1.2–3.3%) and 4.0% (2.6–7.3%), respectively. The overall proportion of nymphs infected with at least one of the studied pathogens is 18.4%. We also observed an increasing relationship between the number of infected nymphs and nymph abundance (Kendall's τ = 0.7556; p < 10^–4^), as shown by the regression analysis in Fig. 3C. The regression predicts that 18.26% of nymphs are infected across the 160 experimental points (40 sites × 4 times).

**Table 2:** Abundance and proportions (%) of nymphs infected with major pathogens, grouped by survey and soil substrate.

Survey	Soil substrate	Nymphs analyzed	BOR (%)	HTRF (%)	ANA (%)
June 2020 (S1)	CL	594	61 (10.3)	23 (3.9)	18 (3.0)
	SA	130	7 (5.4)	1 (0.8)	1 (0.8)
	Total	724	68 (9.4)	24 (3.3)	19 (2.6)
April 2021 (S2)	CL	796	108 (13.6)	17 (2.1)	24 (3.0)
	SA	120	10 (8.3)	2 (1.7)	0 (0.0)
	Total	916	118 (12.9)	19 (2.1)	24 (2.6)
May 2021 (S3)	CL	648	89 (13.7)	11 (1.7)	25 (3.9)
	SA	171	28 (16.4)	8 (4.7)	5 (2.9)
	Total	819	117 (14.3)	19 (2.3)	30 (3.7)
June 2021 (S4)	CL	701	76 (10.8)	7 (1.0)	50 (7.1)
	SA	66	13 (19.7)	2 (3.0)	6 (9.1)
	Total	767	89 (11.6)	9 (1.2)	56 (7.3)
Grand total		3226	392 (12.2)	71 (2.2)	129 (4.0)

Abbreviations: BOR, *Borrelia burgdorferi* sensu lato; HTRF, *Borrelia miyamotoi;* ANA, *Anaplasma phagocytophilum*; CL, clay-limestone area; SA, sandstone area.

### Modeling

Regression analyses allowed a more refined evaluation of the previous results, although significant associations in regression analyses should not be interpreted as causal relationships, especially given the multifactorial nature of this study.

Table 3 shows the fitted, reduced models predicting nymph abundances as a function of the explanatory variables listed in Eq. (1) (“Statistical methods”) These models were calculated for each of the three experimental designs presented in “Statistical methods”. Table 3 also shows the reduced models predicting the abundance of each of the three pathogen families, as well as the total number of pathogens, according to Eq. (2). However, we only considered the first experimental design for these models due to the relatively smaller sample size of ticks that tested positive for pathogens.

**Table 3:** Fitted regression models predicting the abundance of nymphs, the abundance of infected nymphs, and the proportion (logit) of nymphs infected with each of the pathogens BOR, HTRF, and ANA.

Regression model	Dependent variable	Design	Fitted model		Dispersion parameter
Negative binomial	Nymph’s abundance	1	$${{\text{log}}}_{10}\left({\text{nymphs}}\right)=0.7797+1.0892\mathrm{ CL}+\left\{\begin{array}{c}-0.5034 Me\\ -0.8404 O\end{array}\right.+ \left\{\begin{array}{c}0.2517 S2\\ 0.2477 S3\end{array}\right.+ \left\{\begin{array}{c}-1.3724 CL*Me\\ -1.3817 CL*O\\ -0.5239 CL*W\end{array}\right.$$	Equation 3	θ = 1.40
		2	$${{\text{log}}}_{10}\left({\text{nymphs}}\right)=1.0797+0.7337\mathrm{ CL}+\left\{\begin{array}{c}-0.2193 D\\ -0.1733Mix\end{array}+ \left\{\begin{array}{c}0.2966 S3\\ -0.3184 S4\end{array}+\left\{\begin{array}{c}-0.3840 S*S2\\ -0.5157 S*S3\end{array}\right.+\right.\right.\left\{\begin{array}{c}0.5414 CL*S2\\ 0.3241 CL*S3\\ 0.4966 CL*S4\end{array}\right.$$	Equation 5	θ = 3.10
Negative binomial	Infected Nymph’s abundance	1	$${{\text{log}}}_{10}\left({\text{infected}}\right)= 1.1987\mathrm{ CL}-2.1758\mathrm{ O}+ 0.6723\mathrm{ S}3+ \left\{\begin{array}{c}-1.4514 CL*Me \\ -0.6150 CL*W\end{array}\right.$$	Equation 6	θ = 1.35
		2	$${{\text{log}}}_{10}\left({\text{infected}}\right)= 0.5009\mathrm{ CL}-0.5311\mathrm{ Mis}+ \left\{\begin{array}{c}0.6046 S2\\ 0.7016 S3\end{array}\right.$$	Equation 7	θ = 1.49
		3	$${{\text{log}}}_{10}\left({\text{infected}}\right)= 1.0952\mathrm{ CL }+0.6896\mathrm{ S}3-0.4730\mathrm{ S}*{\text{D}}+ \left\{\begin{array}{c}- 0.7056 S*S2\\ -0.5522 S*S3\end{array}\right.$$	Equation 8	θ = 2.73
Logistic	Proportion of infected nymphs	1	BOR:${\text{ln}}\left(\frac{{\text{p}}}{1-{\text{p}}}\right)= -2.3247+0.5719\mathrm{ W}+0.4356\mathrm{ S}3$	Equation 9	
			${\text{HTRF}}:\mathrm{ ln}\left(\frac{{\text{p}}}{1-{\text{p}}}\right)= -3.3730-1.0604\mathrm{ S}4$	Equation 10	
			${\text{ANA}}:\mathrm{ ln}\left(\frac{{\text{p}}}{1-{\text{p}}}\right)= -3.6138+1.0724\mathrm{ S}4$	Equation 11	
			$${\text{Total}}:\mathrm{ ln}\left(\frac{{\text{p}}}{1-{\text{p}}}\right)= -1.7413+0.3968\mathrm{ W}+\left\{\begin{array}{c}0.3139 S3\\ 0.3281 S4\end{array}\right.$$	Equation 12	

Abbreviations: BOR, *Borrelia burgdorferi* sensu lato; HTRF, *Borrelia miyamotoi*; ANA, *Anaplasma phagocytophilum;* CL, Clay-Limestone; Me, Meadow; O, Orchard; W, Wetland; Mis, Missing; S, Stable; D, Deciduous; S1, S2, S3, S4, the four surveys (see Table 2). Asterisks (*) indicate interactions between factors.

The regression coefficients of all fitted models are statistically significant at the 1% probability level. Confidence intervals for regression coefficients and odds ratios are given in Supplementary Material S2.

As an example, according to the first fitted model in Table 3, the estimated abundance of nymphs in “Clay-Limestone-Wetland” sites sampled during survey 2 would be determined as: log_10_ (nymphs) = 0.7797 + 1.0892 × (+ 1) + 0 + 0.2517 × (+ 1) − 0.5239 × (+ 1) × (+ 1) = 1.5967, which equates to 39.51 nymphs per 200 m^2^. Note that this model output is very close to the observed mean number of 40 nymphs per 200 m^2^.

Similarly, the total proportion of infected nymphs in wetlands during sampling period S4 would be calculated as ${\text{ln}}\left(\frac{{\text{p}}}{1-{\text{p}}}\right)=$ − 1.7413 + 0.3968 × (+ 1) + 0.3281 × (+ 1) = − 1.0164, which equates to an infection rate of 26.57%. On other soils and during sampling periods S1 or S2, this proportion was estimated at 14.91%

All the environmental factors we considered (i.e., soil substrate, soil occupation, dynamics, forest stands, and survey month/year) were included in the fitted models shown in Table 3, either as main effects or in interactions. These fitted models can be used for both predictive and explanatory purposes. For the most part, the model outcomes supported the results of our descriptive analyses.

### Nymphs and infected nymphs

We observed a significant effect of the survey period on the abundance of nymphs and of infected nymphs: surveys S2 and S3 (April 2021 and May 2021) were characterized by higher total nymph abundances than surveys S1 (June 2020) and S4 (June 2021) (2–5 times more, according to Eqs. 3, 6, and 7). There was also a clear "soil substrate" effect: model-estimated nymph abundances were higher on CL than SA (up to 16 times more). However, the soil substrate effect was modulated by significant interaction effects with soil occupation (Eqs. 3 and 6) and the survey period (Eq. 5).

In our models for *I. ricinus*, we also observed a significant "soil occupation" effect: the density of nymphs and infected nymphs was higher in wetlands, and especially in forests, than in other habitats. Across all the habitat types we considered, nymph abundance was lowest in orchards, followed by meadows, wetlands, and then forests (Eqs. 3 and 6). The abundances of nymphs and of infected nymphs were also up to 3 times higher in current wetlands than in former wetlands that had since assimilated to meadows (Eqs. 4 and 7).

There was no overall effect of the "dynamic" factor on nymph abundance, except for a significant effect within wetlands, where missing wetlands showed lower abundance of nymphs, infected or not (minus 65–70%, according to Eqs. 4 and 7). In forests, there were significant interactions between the level "stable" and the sampling period (Eqs. 5 and 8). These interactions resulted in a relative decrease in nymph abundance during sampling periods 2 and 3 (when overall nymph abundance was highest) in stable forests compared to recent forests. This sampling period-specific decrease was more prominent in deciduous forests (Eq. 8). In other words, tick nymphs were more abundant in current vs. former wetlands and in recent vs. stable forests, and the latter phenomenon was particularly observable during the April–May study period. We additionally noted that areas of recent forest colonization, which are characterized by herbaceous shrub and pioneer tree species, supported a higher density and diversity of vegetation than older, stable forests.

Within forest ecosystems, we also observed a significant "stand" effect: nymph abundance depended on the forest type, with deciduous forests having the fewest ticks and coniferous forests having the most (plus 67%, Eq. 5). This pattern was less pronounced for the abundance of infected nymphs, which were less abundant in stable deciduous forests but more consistent between mixed and coniferous forests (Eq. 8).

In forests, the combinations of factor levels corresponding to the estimated maximum and minimum nymph abundance were CL-Co-R-S3 (271.71 nymphs/200 m^2^) and SA-D -S-S2 (2.99 nymphs/200 m^2^) respectively (see Eq. 5), which is consistent with the observations of the descriptive approach (255 nymphs/200 m^2^ and 1 nymph/200 m^2^, respectively). As for the infected nymphs, the corresponding combinations were CL-Mix or Co-R-S3 (60.93 nymphs/200 m^2^) and SA-D-S-S2 (0.07 nymphs/200 m^2^), respectively (see Eq. 8). Again, these estimates were consistent with the observed counts of 44.62 (after adjusting for the number of nymphs analyzed/nymphs enumerated) and 0, respectively, and allowing for the variability inherent to the negative binomial model.

### Proportion of infected ticks

The proportion of ticks infected with any of our three focal pathogens (BOR, HTRF, ANA) varied with time and with soil occupation (Fig. 3A,B). Based on our corresponding regression results (Eq. 12), the estimated proportion of ticks infected with any pathogen was highest (26.57%) in wetlands during survey S4 and lowest (14.91%) in other habitats during surveys S1 and S2. When we modeled each pathogen separately, we found that the proportion of ticks infected with BOR was highest in wetlands during survey S3 (21.13%) (Eq. 9), whereas during survey S4, the proportion of ticks infected with ANA reached its maximum (7.30%) (Eq. 11) and the proportion infected with HTRF reached a minimum (1.17%) (Eq. 10).

### Links between nymph abundance, fauna presence and soil physico-chemical characteristics.

**Figure 4:**
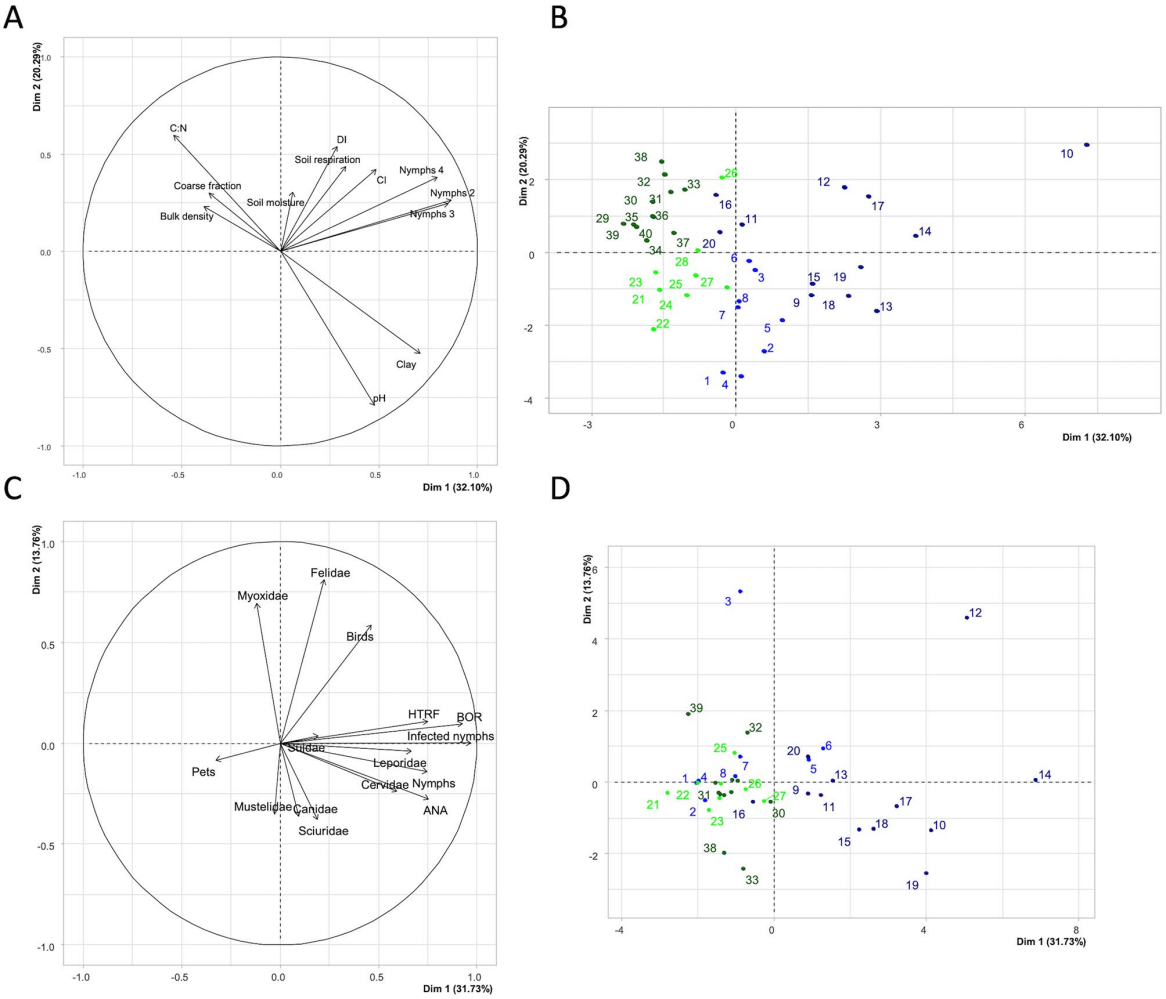
(**A**,**B**) PCA of nymph abundance, pathogen abundance, camera trap-based measures of faunal diversity, and soil physicochemical characteristics. (**A**) Variables; (**B**) Sites (parcels). (**C**,**D**) PCA of wildlife and pathogen abundances. (**C**) Variables; (**D**) sites (parcels). The parcels associated with the point numbers in panels B and D are shown in Table 1. Points corresponding to clay limestone-based and sandstone-based soils are in blue and green, respectively, and forested patches are darkly colored. Abbreviations: BOR, *Borrelia burgdorferi* sensu lato; HTRF, *Borrelia miyamotoi*; ANA, *Anaplasma phagocytophilum*; CI, contact index; DI, diversity indicator.

We used principal component analysis (PCA) to investigate the multivariate associations among (i) nymph abundances observed during the last three surveys, (ii) camera trap-based indicators of wildlife presence, and (iii) physico-chemical characteristics of the soils (C:N, coarse fraction, bulk density, soil moisture, respiration, clays and pH, as reported in Supplementary Material S1. These results are shown in Fig. 4A,B for both the variables and the 40 sampling sites. The first and second components of the PCA explain 32.10% and 20.29%, respectively, of the variation in the data. As with our first PCA, Component 1 distinguished the parcels according to their soil substrate. Component 2 separated sites located in forests (top of Fig. 4B) and sites from other soil occupations (bottom of Fig. 4B).

With respect to the PCA-based relationships among variables (Fig. 4A), we observed positive correlations among nymph abundance, the presence of wildlife, and soil respiration. Conversely, there was a strong inverse relationship between the soil C:N ratio and each of soil pH and clay content. Parcels located in CL soils (right of Fig. 4B) were characterized by a higher density of nymphs, whereas parcels in SA soils (left of Fig. 4B), especially forested patches, were characterized by high soil C:N, low pH, and low clay content.

In addition to the PCA of Fig. 4A,B, a correlation analysis highlighting the most relevant and also statistically significant associations between nymph abundance, pathogen abundance, indicators of faunal presence (i.e. contact index [CI] and diversity indicator [DI]) and soil characteristics can be found in the Supplementary Material S3.

Figure 4C,D show the PCA-based links among the abundances of tick nymphs and their associated pathogens and the abundances of animal families identified via camera traps: Cervidae (mainly roe deer), Suidae (wild boars), Canidae (foxes), Felidae (wild cats), Mustelidae (martens, badgers), Myoxidae (dormice, field mice), Sciuridae (squirrels), Leporidae (hares). Here Pets includes domestic animals (dogs, cats, chickens) mainly identified in orchards or in the vicinity of houses, birds include all bird species. Component 1 of this PCA explained 31.73% of the total variation in the data and was characterized by positive correlations among the abundance of nymphs (infected or not), the abundance of the three pathogens, and the abundance of certain faunal families including birds, Cervidae and Leporidae (Fig. 4C). Component 2 explained 13.76% of the variation and, as with the previous PCA analysis, discriminates parcels by their soil substrate; parcels in CL-based soils were associated with higher nymph abundances (Fig. 4D).

Table 4 summarizes the three main results of our correlation analysis between the abundance of nymphs or associated pathogens and the different wildlife families we considered. First, we observed decreasing relationships between domestic animals and the abundances of nymphs, BOR, and ANA.

**Table 4:** Correlation analysis (Kendall’s τ) between nymphs, pathogens and wildlife.

	Nymphs	Infected nymphs	HTRF	ANA	BOR	Genospecies^a)^
Birds	τ = 0.2928 p = 0.0089**	τ = 0.3733 p = 0.0010**	τ = 0.2905 p = 0.0194*	τ = 0.2588 p = 0.0322*	τ = 0.4025 p = 0.0004**	*B. afzelii:* τ = 0.4823; p = 0.0003**
Canidae	τ = 0.1005 p = 0.3870	τ = 0.1321 p = 0.2635	τ = 0.0554 p = 0.6682	τ = 0.1480 p = 0.2383	τ = 0.1234 p = 0.2996	
Cervidae	τ = 0.3403 p = 0.0022**	τ = 0.2954 p = 0.0090**	τ = 0.1704 p = 0.1679	τ = 0.2820 p = 0.0188*	τ = 0.2527 p = 0.0265*	*B. garinii:* τ = 0.3793; p = 0.0057** *B. b ss:* τ = 0.3391; p = 0.0211*
Leporidae	τ = 0.4671 p = 0.0001**	τ = 0.5423 p < 10^–4^**	τ = 0.2153 p = 0.1156	τ = 0.5039 p = 0.0001**	τ = 0.5457 p < 10^–4^**	*B. afzelii:* τ = 0.4719; p = 0.0011** *B. garinii:* τ = 0.6070; p < 10^–4^ **
Mustelidae	τ = 0.0504 p = 0.6721	τ = 0.0397 p = 0.7432	τ = 0.0051 p = 0.9692	τ = 0.1206 p = 0.3488	τ = 0.0074 p = 0.9515	*B. b ss:* τ = 0.3445; p = 0.0287*
Myoxidae	τ = 0.0162 p = 0.8922	τ = -0.0090 p = 0.9409	τ = 0.0680 p = 0.6096	τ = 0.0067 p = 0.9589	τ = 0.0258 p = 0.8332	*B. garinii:* τ = -0.3262; p = 0.02538*
Pets	τ = − 0.2683 p = 0.0299*	τ = − 0.2390 p = 0.0571	τ = − 0.0622 p = 0.6509	τ = − 0.3304 p = 0.0134*	τ = − 0.2483 p = 0.0497*	*B. afzelii:* τ = − 0.3323; p = 0.0278*
Sciuridae	τ = 0.2250 p = 0.0585	τ = 0.2191 p = 0.0699	τ = 0.1399 p = 0.2901	τ = 0.3752 p = 0.0035**	τ = 0.1791 p = 0.1414	*B. b ss:* τ = 0.4017; p = 0.0099**
Suidae	τ = 0.1325 p = 0.2439	τ = 0.1311 p = 0.2567	τ = − 0.0095 p = 0.9401	τ = 0.1272 p = 0.3002	τ = 0.0882 p = 0.4485	
Wild felidae	τ = 0.2019 p = 0.1028	τ = 0.2439 p = 0.0526	τ = 0.2196 p = 0.1108	τ = 0.1078 p = 0.4203	τ = 0.2678 p = 0.0346*	

Abbreviations: BOR: *Borrelia burgdorferi* sensu lato; HTRF: *Borrelia miyamotoi;* ANA: *Anaplasma phagocytophilum.* B. b ss: *Borrelia burgdorferi* sensu stricto. *Significant at the 5% probability level, **significant at the 1% probability level. ^a^Ties did not allow for the calculation of exact p-values.

Second, we observed that the abundances of nymphs and their associated pathogens were positively related with observations of Cervidae, Leporidae, and birds, though only birds were positively related with HTRF. Birds were the only faunal group correlated with all three pathogenic microorganisms considered in our study.

Finally, we observed increasing relationships between Sciuridae and ANA and between wild Felidae and BOR.

**Figure 5:**
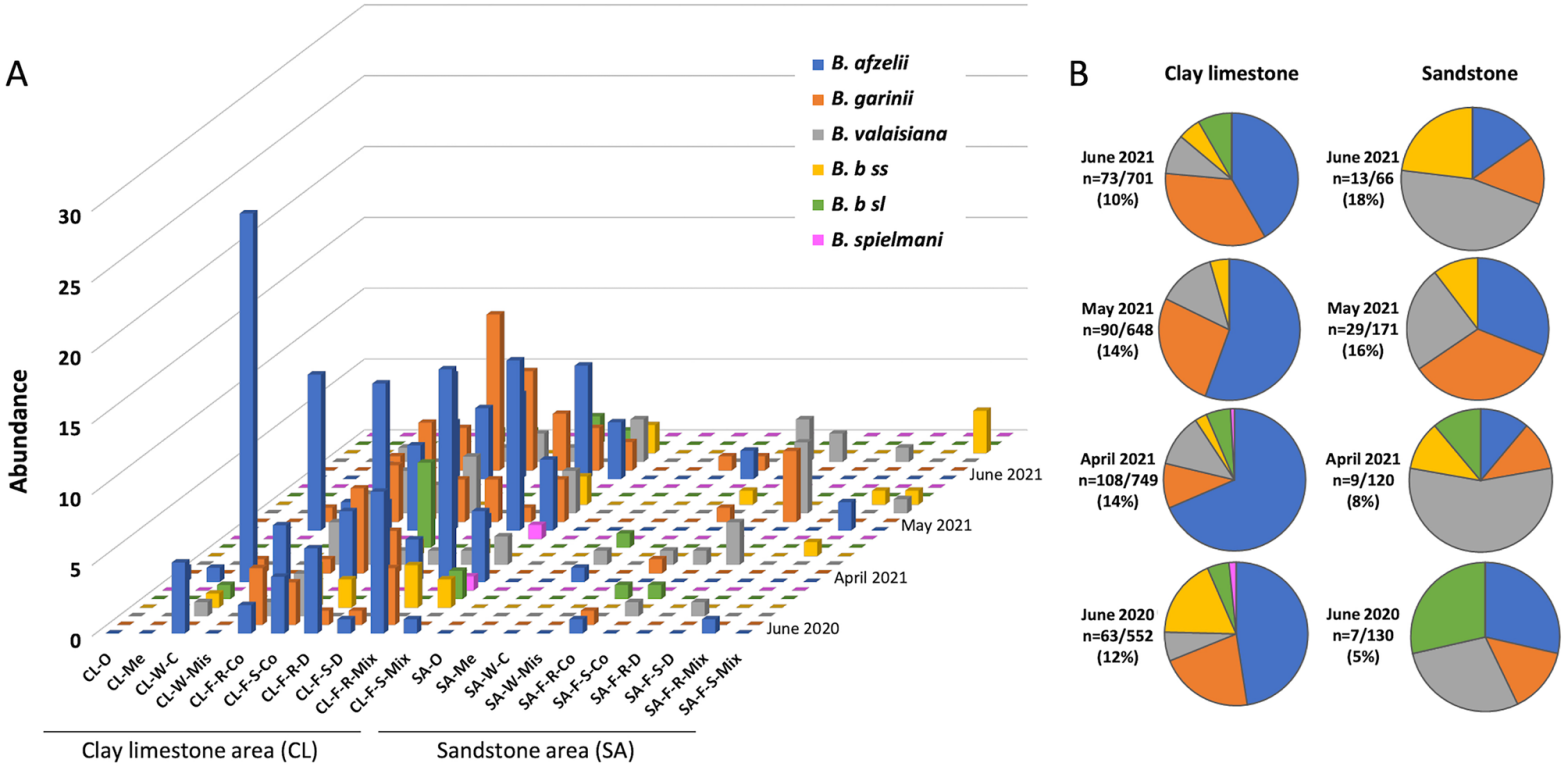
(**A**) Abundances of different genospecies of *Borrelia burgdorferi*: *B. afzellii, B. garinii, B. valaisiana, B. burgdorferi (B. b) s.s., Borrelia burgdorferi *sensu lato* (B. b sl),* and *B. spielmanii*. Abundances are shown separately for the different survey periods. (**B**) The abundance of the *Borrelia burgdorferi sl* genospecies expressed as a percentage of positive samples per site and per collection date in different soil types. *CL* clay-limestone, *SA* sandstone, *O* orchard, *Me* meadow, *W* wetland, *C* current, *Mis* missing, *F* forest, *R* recent, *S* stable, *Co* conifers, *D* deciduous, *Mix* mixed. Code for sampling sites are given in Table 1.

In our study, we mainly detected *B. afzelii* in clay-limestone (CL) sites but *B. valaisiana* and *B. garinii* in sandstone sites (SA) (Fig. 5A,B). More specifically, we found that the presence of *B. afzelii* was positively correlated with the abundance of birds and Leporidae*.*

The presence of *B. garinii* was also positively correlated with the abundances of Cervidae and Leporidae. The presence of *B. burgdorferi* s.s. was correlated with the abundances of Cervidae, Sciuridae, and Mustelidae.

### Machine learning analysis results for nymph and pathogen abundance

**Figure 6:**
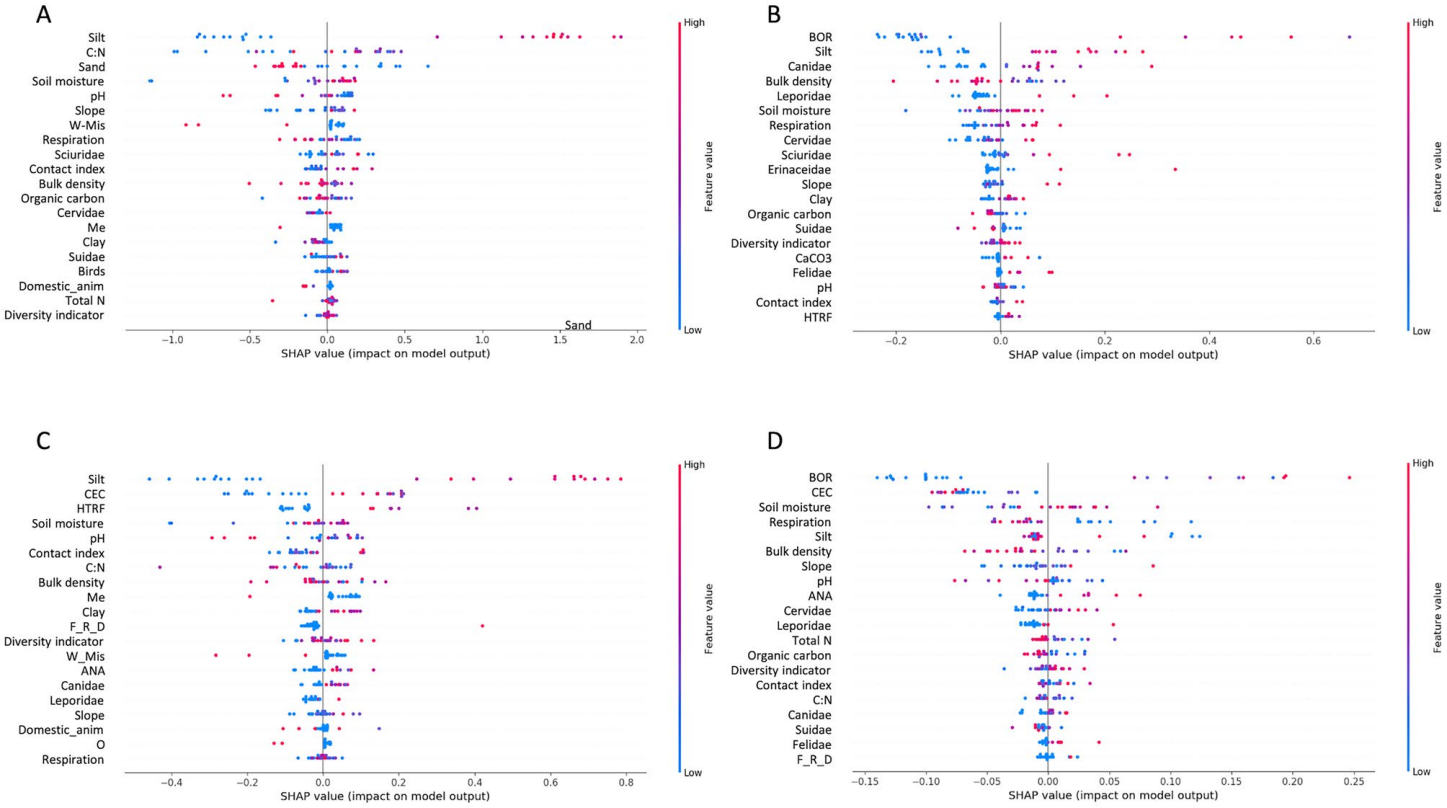
Machine learning-based classification of the importance of different input variables on the abundance of (**A**) tick nymphs, (**B**) ANA pathogen, (**C**) BOR pathogen, and (**D**) HTRF pathogen. Abbreviations: BOR, *Borrelia burgdorferi* sensu lato; HTRF, *Borrelia miyamotoi;* ANA, *Anaplasma phagocytophilum*; CL, clay-limestone area; SA, sandstone area; O, orchard; Me, meadow; W, wetland; C, current; Mis, missing; F, forest; R, recent; S, stable; Co, conifers; D, deciduous; Mix, mixed. Code for sampling sites are given in Table 1.

Given that several explanatory variables participated in two-way interactions with each other, we completed our correlation analysis using a machine learning approach to identify the best variables influencing the abundances of nymphs and their associated pathogens. Based on RMSE and R2 estimation, the best model ranking is presented in Supplementary Material S4. The XGB Regressor was the best model among the seven models we tested. We therefore used the XGB Regressor model to determine the explanatory variables that most strongly drove tick abundance. Figure 6A shows that the most important explanatory variables for the abundance of nymphs were those related to soil characteristics. Specifically, the most important input variable was the presence of silts, insofar as silty soils harbored more ticks and low-silt soils supported fewer ticks. After silt, sand percentage and soil moisture were important input variable affecting the abundance of nymphs. Clearly, tick abundances were lower in soils with a high percentage of sand which are more related to sandstone substrate than on clay-limestone substrate. Soil moisture was also an important driver of tick abundances, with higher moisture contents favoring more *I. ricinus* nymphs. The influence of the C:N ratio on tick abundance, although important, is not clear to decipher. Indeed, both high and low C:N values can have a positive or negative effect on tick abundance, probably by interfering with other factors. The effect of pH also seems complex, since some higher pH values have a negative impact on tick abundance.

Tick abundance was highly variable in missing or former wetlands (W_Mis in Fig. 6), presumably because of the divergent nature of the ecosystems that overtake former wetlands (e.g., meadows vs. wastelands with shrubs). Soil microbial respiration was one of the most important biological parameters predicting tick abundance. With respect to biodiversity-based predictors, the presence of Sciuridae were the strongest driver of tick abundance. Specifically, a high abundance of Sciuridae, along with a high abundance and/or contact index for birds or deer, had a positive effect on the tick abundance.

We further tested the same seven machine learning models for their ability to predict the abundance of the three tick-borne pathogens we studied. Model performance results for the ANA, BOR and HTRF pathogens are presented in Supplementary Materials S5, S6, and S7, respectively. The XGB Regressor model was once again the best model for predicting the abundance of all three pathogens, so we used this model to determine the relative importance of different explanatory variables for predicting the abundance of each pathogen. Results of these models are shown in Fig. 6B–D.

This second set of models showed that soils with a high silt content, as well as the presence of BOR, Canidae, Cervidae, Sciuridae, or even Leporidae, positively affected the abundance of the ANA pathogen. This pathogen was also mainly observed in forests with higher soil moisture and lower soil aerobic respiration (Fig. 6B). The effect of different explanatory variables on the abundance of BOR-type pathogens was more nuanced (Fig. 6C): silt-rich soils had a clear positive effect on the abundance of BOR pathogens, and cation exchange capacity and the contact index and diversity indicator had a lesser but still positive effect. Increased soil moisture, lower soil respiration, a higher abundance of BOR or ANA pathogens were all positively related to the abundance of HTRF-type pathogens (Fig. 6D). Overall, the machine learning-based analyses of our data confirmed that soil type has a strong effect on the abundance of *I. ricinus* and the prevalence of three major tick-borne pathogens.

## Discussion

In this study, our experimental design enabled us to clearly answer our initial questions relative to the influence (1) of soil substrate, (2) land occupation, (3) two centuries temporal dynamics and (4) forest stands on tick abundance.

Moreover, we simultaneously explore a large number of tick explanatory variables most associated with the abundance of human bacterial pathogens. This approach, using artificial intelligence, enabled the identification of the best predictors among numerous variables. Although individual effects have previously been reported for most variables, their interactions have rarely been considered in a comprehensive study.

We confirmed the seasonal effects^15,24^ and infection prevalence in Northeastern France^23,39^. However, the prevalence of the *B. burgdorferi* s.l. is lower at higher latitudes, such as in Denmark^40^, thus suggesting that climate may be an important driver of pathogen abundance.

The effects of soil on tick abundance have been reported for *I. scapularis*^41,42^. For *I. ricinus*, Goldstein et al.^24^ have reported lower tick abundance on clay, and a favorable effect of moder on tick abundance. Specifically, sandy soils with low water retention have been found to be less favorable for ticks than silt-rich soils, which have a high organic matter content and efficiently retain moisture. Indeed, a soil water capacity effect has been described^40^.

The highest probability of ticks has been reported in forested areas or areas with vegetation (> 1 m) and permanent leaf litter^16,43^, and in ecotones between forests and arable fields^8,19^. This was the case in some sampled orchards, meadows, and wetlands located near forested areas in the CL ecosystems (site numbers 1–6). This ecotone effect might also explain the presence of BOR-infected nymphs in these specific sites. Forest environments are characterized by abundant leaf litter, which maintains favorable moisture conditions for the desiccation-sensitive *I. ricinus*^15^. Forest composition, diversity, and structure can exert diluting or amplifying effects on the abundance of ticks and other tick-borne pathogens, thereby suggesting that forests can have multifactorial effects^44^. According to Ehrmann et al.^45^, food, shelter, and abundance of micro- and macro-habitats can serve as predictors of tick abundance. The higher abundance of nymphs and infected nymphs in transitional environments, such as recent forests, was attributed to more abundant and/or more diverse *I. ricinus* hosts in these habitats. Recent forests are unmanaged, difficult to penetrate and with virtually no human presence, making them ideal refuges for wildlife which may increase host density in such area. Indeed, clear-cutting forests has been reported to decrease the abundance of nymphs and infected nymphs^46^. Forest tropisms for ANA have occasionally been reported^47,48^.

Forest stand effects have been poorly documented in the literature, although a recent study has reported a positive effect of conifers on the abundance of tick larvae^44^. The density of questing nymphs, the prevalence and the density of infected nymphs have often been associated with stands of deciduous trees (mainly oaks) and with the local abundance of deer^49^. However, the effect of forest stands described in the literature should be treated with caution, as tick abundance may differ depending on whether areas with easier access for sampling, such as pathsides, are considered. This mainly concerns coniferous forests, which are sometimes difficult to penetrate. Altogether, our results concerning recent forests and those concerning the nature of forest stands raise the question of the effect of forest management on tick abundance.

The combined effects of factor levels identified herein reinforced the previously described need for a more dynamic view emphasizing spatial and temporal interactions at multiple scales^50^.

The survey effect suggested that the factors driving the prevalence of the different microorganisms varied, presumably as a function of the host dynamics for each microorganism^15^ and of humidity and temperature parameters.

Domestic animals are a sign of anthropogenic influence, which is unfavorable to the main vectors of nymph dissemination (i.e., wildlife such as Cervidae) and therefore to the dissemination of their associated pathogens. Birds are known reservoirs of *B. burgdorferi* s.l.^48^. Leporidae (hares) are also known hosts for all three tick life stages, as well as for *B. burgdorferi* s.l.^19,51^. Small and large mammals (roe deer and wild boar) are well-known hosts of ANA^19,27,52^. Ungulates are amplifying hosts for ANA but dilution hosts for BOR^48^, because they are not a reservoir for *B. burgdorferi* s.l. The strong association between *B. burgdorferi* s.l. and specific hosts is largely a function of complement evasion^53^. Biodiversity can be seen to decrease the prevalence of ticks infected with *B. burgdorferi* s.l. by increasing the abundance of non-reservoir hosts.

In their dilution effect model, LoGiudice et al.^54^ have found that vertebrate biodiversity buffers the human risk of exposure to tick-borne pathogens, and have specifically considered Sciuridae to be a dilution host for BOR, characterized by high tick loads and high population density, but limited ability to act as a BOR reservoir. LoGiudice et al.^54^ did not find any relationships between nymph or pathogen abundance and the other investigated wildlife species (Suidae, Myoxidae, Canidae, Mustelidae). However, whether these species are not tick reservoirs or these results were false negatives due to the limited amount of data available for these species is unknown. For example, the latter is probably true for small mammals, because they have been experimentally demonstrated to be reservoir hosts for *B. afzelii*, *B. burgdorferi* s.s., *B. spielmanii*, and *B. miyamotoi*^19,55^. The results for Suidae are also likely to be a statistical artifact, because wild boar has been reported to contribute to the epidemiology of tick-borne ANA^56^, although to a lesser extent than ungulates^57^. However, the dilution effect of biodiversity on the abundance of infected ticks is uncertain^27^ and seems to depend on the specific composition of the communities under consideration^58^, biotic interactions within these communities^59^ and the spatial scale^60^.

Five genospecies of BOR pathogens (*B. garinii*, *B. afzelii*, *B. spielmanii*, *B. valaisiana*, and *B. burgdorferi* s.s.) are common in Europe and can infect humans^30^. The circulation of these genospecies depends on the abundance of their respective vertebrate hosts. Until recently, birds were not considered reservoirs for *B. afzelii*, because their immune systems have been demonstrated to be lethal against this species^53,61^. However, some birds can serve as reservoirs for *B. afzelii*^56^ and *B. garinii*^62^. In our results, the presence of *B. burgdorferi* s.s. correlated with the abundance of Cervidae, Sciuridae, and Mustelidae, all of which are previously described reservoirs for this species^62^. Among the Cervidae, roe deer are a well-documented host for *Ixodes* ticks but are not considered a reservoir for *B. burgdorferi* s.l.^25,27^.

In the present multifactorial study, owing to the large number of variables analyzed, we used artificial intelligence to identify the best predictors of tick abundance and tick infection with specific human pathogen agents. This new method of data analysis reinforced the importance of soil parameters as strong drivers of tick abundance. Particularly striking is the abundance of ticks in areas with clay-limestone soil compared to areas with sandstone and acidic soil. Clay-limestone soils are more suitable for agriculture than sandstone soils, which are more acidic and nutrient-poor and on which only the forest can grow. Clay-limestone soils also hold water better than sandstone, which is a factor favoring tick abundance. Moreover, it should be noted that in agricultural areas, hunters protect cereal crops (wheat and maize in particular) by artificially feeding the game with maize, especially in forest plots. This practice could also explain the development of a certain fauna, notably roe deer and wild boar, but also birds and rodents in agricultural area. In addition, in these agricultural areas, contact between ticks and hosts is probably increased by these forest patches, and the circulation of infectious agents facilitated.

To conclude, we found that both soil- and host-related factors are essential for predicting the presence and abundance of potentially pathogenic microorganisms in the local tick population. Drivers of pathogen abundance varied among the pathogens studied, and machine learning algorithms helped us isolate the effects of interacting factors.

We recommend extending the approach used herein to include other soil typologies and/or geographical regions, to confirm how soil type may drive the abundance of ticks and tick-borne diseases. Future studies could also explore how different forest management practices and/or biodiversity protection policies exacerbate or mitigate the human health risks of tick-borne diseases.

## Methods

### Tick sampling strategy

We selected two study sites within the Pays de Bitche region in northeastern France. The two sites were approximately 20 km apart and had similar altitude and climate but different geological substrates. The first site, the commune of Eguelshardt, is located on the Vosges sandstone substratum, dating from the Triassic period and locally covered with alluvium. These soils are sandy, relatively acidic Podzosols that are locally classified as Brunisols or Reductisols^63^. Our second site, the commune of Achen, is also located on a Triassic substrate; however, this substrate is of a clay-limestone nature and can be locally covered with alluvium or silt. These soils have a finer texture and are less acidic than soils developed from sandstone. They are dominated by Calcosols and Calcisols, with local Fluviosols on alluvium^63^ (Supplementary Material S8).

At each site, we compared the tick populations among the following habitats: orchards, meadows, current wetlands, former wetlands, recent forests, and stable forests. We used maps of these two areas from the period 1758–1812 (either the “topogeographic” atlas established in 1758 on a scale of 1/4300 and/or the Napoleonic cadastre) (Rochel, personal communication) to identify stable *versus* recent forests as well as former wetlands. Comparisons between recent and stable forests were made separately for coniferous, deciduous, and mixed forests. For each of our 10 habitat types, we selected duplicate locations at each of our two study sites. GPS coordinates of our 40 total sampling sites, as well as their soil characteristics are reported in Supplementary Material S9.

This experimental design allows us to test the effects of four different factors that may affect tick or pathogen abundance: (1) soil substrate (clay limestone *vs.* sandstone acid); (2) habitat type (orchard, meadow, wetland, or forest); (3) time (current *vs.* former for wetlands, recent *vs.* stable for forests); and (4) forest type (coniferous, deciduous, or mixed). This analysis scheme is also visualized in Fig. 1. Each combination of these four levels of analysis included two replicate parcels, and all 40 sampling sites were analyzed during each of four sampling surveys (June 2020, April 2021, May 2021 and June 2021). To complete information, sampling surveys were initially scheduled in April, May and June 2020, but due do covid the April and May 2020 surveys were not authorized.

### Vegetation characterization

In June 2020, we performed a floristic inventory of all 40 plots, except for orchard plots because the herbaceous layer is poor. This inventory is presented in Supplementary Material S10.

### Tick collection

Nymph ticks were collected from sample sites following previously described methods^24^. In brief, we dragged a 1 m^2^ white terry cloth over the underlying vegetation. After covering a distance of 10 m (i.e., an area of 10 m^2^), the cloth was turned over and any attached ticks were collected and transferred into a tube. Twenty drags were performed at each site for each survey. Live ticks were brought back to the laboratory and frozen at − 20 °C until DNA extraction.

### PCR analyses for detection of Ixodes ricinus-borne pathogens

Total tick DNA was extracted using ammonium hydroxide. To detect tick-borne bacterial pathogens in *I. ricinus* nymphs, we then ran multiple PCR and qPCR-based assays following previously described methods^64^. Briefly, a first *Borrelia* PCR targeting the *flagellin b* gene was performed, followed by a second real time PCR for positive samples, using specific fluorescent hybridization probes for each *Borrelia* species. We tested a maximum of 60 ticks per sampling site per survey, when possible, to assess the relative prevalence of each microorganism.

The presence of *B. burgdorferi* s.l. DNA in tick extract was determined with real-time PCR using a primer and two Taqman® probes targeting the conserved region of the *flagellin b* gene. All reactions were performed and analyzed on a CFX OPUS 96 (Biorad). To determine the genotype of any samples that tested positive for *Borrelia*, a second real-time PCR typing assay (LightCycler® 2.0 [Roche]) was performed on each positive sample. These reactions used the same primers as the first PCR assay but included specific fluorescent hybridization probes (10 FRET probes and 1 TaqMan® probe) that are specific for *B. burgdorferi* s.s., *B. garinii/B. bavariensis*, and *B. afzellii*^20^. The melting temperature (Tm) of each probe pair was specific for each *Borrelia* species. In separate reactions, *A. phagocytophilum* and relapsing fever *Borrelia* were detected with real-time PCR assays targeting the *msp2/p44*^65^ and *B. miyamotoi flagellin* genes^66^, respectively.

### Soil analysis

We sampled the organo-mineral surface horizon of the soil at each site in June 2020. Soil bulk density was determined in the field using the core method^67^ by relating the mass of dry soil sampled with a cylinder to its volume. All other analyses were performed on the air-dried, fine-earth fraction obtained by sieving the soil through a 2 mm sieve. As part of the sieving process, we also calculated the mass percentage of the coarse fraction. Soil pH was measured in a 1:5 solution of soil: water. Organic carbon and total nitrogen content were analyzed by dry combustion (Dumas method ISO 10694). CaCO_3_ content was determined by measuring the volume of CO_2_ released after 4 M HCl treatment (NF ISO, 10693, 1995). Cation exchange capacity (CEC) was measured using the cobaltihexamine method (NF X 31–130). Particle size analysis (ISO 11277) was performed using the pipette method^68^ and wet sieving to recover the clay, silt, and sand fractions. The main soil characteristics at each site are reported on Supplementary Material S1.

We determined soil moisture content on each of our four sampling days by the weight loss between the sample measured before and after drying. At each forested site, we collected soil litter in 25 × 25 cm quadrats and then dried samples at 80 °C for three days.

Soil microbial activity was assessed using soil respiration assays performed for four independent samples in MicroResp™ microplates (James Hutton Limited, Dundee, UK)^69^. Microplates were used as recommended by the manufacturer to determine CO_2_ production. Soil samples were dried at room temperature, sieved through a 2.0 mm stainless steel sieve, and then stored at 4 °C until respiration assays were performed. Prior to analysis, soils were humidified to 50% of their water-holding capacity over three days at 25 °C. CO_2_ production was then measured from 400 mg of each sample during a six-hour incubation at 25 °C. The detection plates contained a red cresol solution (12.5 µg/ml in 150 mM KCl and 2.5 mM NaHCO_3_) dissolved in 1% agar. The red cresol solution changes from pink to yellow as the pH decreases due to carbonic acid production, allowing for colorimetric analysis of total respiration. For each sample, we measured absorbance at 570 nm (A_570_) on a SAFAS Spectrophotometer at the beginning and end of the six-hour incubation. Absorbance data for each sample were normalized by dividing the A_570_ at six hours by the A_570_ at time 0 and then multiplying the result by the overall mean A_570_ reading at time 0 across all samples. The normalized A_570_ at time zero was deduced from the normalized A_570_ at time 6 h. All respiration results are reported per g of dried soil.

### Fauna analysis

We placed a camera trap (BOLYGUARD BG590-24MP) on each plot to loosely evaluate the activities of mammals and birds. The traps were used in motion-triggered, 20-s video mode, and traps remained active for one month for each of the April, May, and June 2021 collections. From the camera trap data, we calculated three measures of animal activity: a presence/absence index, a contact index (CI), and a diversity indicator (DI). For the presence/absence index, we recorded either the presence or absence of ten different wild mammal families (Cervidae, Suidae, Canidae, Felidae, Mustelidae, Erinaceidae, Myocastoridae, Myoxidae, Sciuridae and Leporidae), as well as birds and pets (cats, dogs, and hens). The contact index was determined as the total number of individual animals that could be observed on the photo traps during the one-month observation period; if the same individual passed in front of the camera several times, each occurrence was recorded as a new observation. Despite some inherent biases in this approach, the contact index nevertheless provides an overall idea of the abundance of fauna at each site. Finally, the diversity indicator was a single measurement calculated based on the number of families detected on each plot.

### Statistical methods

The issue addressed in the present work is multifactorial, involving numerous variables that may be correlated with one another. There is no single statistical technique capable of simultaneously answering all our questions. Consequently, several complementary approaches were needed to achieve our objectives.

Thus, in order to obtain a clear picture of the importance and role of explanatory variables on nymph and pathogen abundance, we used (i) descriptive techniques (principal component analysis and correspondence analysis), which suggest, by visualizing them, links between variables, (ii) inferential statistics (hypothesis testing, regression analysis), which aim to establish links or conclusions in a probabilistic context, and (iii) machine learning (ML) methods, which take advantage of the computing power of computers. However, the ML approach has a limited explanatory capacity, and is therefore no substitute for the other statistical methods. Only the combined use of these complementary approaches can give us a reliable and comprehensive view of the phenomena under study.

Correspondence analysis (CA) was used to visualize relationships between pathogen abundance and soil occupation. Principal component analysis (PCA) was used three times in this work, notably to examine associations between nymph abundance and, respectively, the different habitat types, the physicochemical characteristics of the soils and the fauna.

With regard to inferential statistics, the non-parametric Friedman test with blocks on the parcels was carried out to test whether nymph abundance differed significantly between the four surveys. Kendall rank correlation coefficient was used to assess monotonic ordinal associations between pairs of variables. The chi-square test was performed to test whether (i) the relative abundance of the three pathogens was the same between the four sampling surveys, (ii) the overall proportion of infected nymphs was stable between surveys, and (iii) the abundance of the three pathogens varied according to soil occupation.

We modeled the abundance of tick nymphs and associated pathogens using negative binomial regression, which is particularly well-suited to over dispersed count data. The parameters µ and θ of the negative binomial distribution, denoted NB (µ, θ), represent the mean and dispersion (shape) of the distribution, respectively. As the value of the dispersion parameter θ decreases, the among-sample variability of the counts increases. The reference model that we used to study the variation in the abundance of nymphs or infected nymphs was expressed by the following link function:

1 $${\text{log}}_{{{1}0}} \mu \, = {\text{ f}}\left( {{\text{soil substrate}},{\text{ soil occupation}},{\text{ dynamics}},{\text{ forest stands}},{\text{ survey}}} \right)$$

In Eq. (1), the logarithm of the mean (µ) of the nymph counts was expressed as a linear function of the explanatory factors listed in Fig. 1 including sampling campaign. We also included all possible two-way interactions between the five factors listed in Eq. (1), which were characterized by 2, 4, 2, 3, and 4 levels, respectively.

We modeled the proportion (p) of nymphs infected with pathogens using logistic regression. For this purpose, the left-hand side of Eq. (1) was replaced by the logit of p.

2 $${\text{ln}}_{{}} \left( {{\text{p }}/ \, \left( {{1} - {\text{p}}} \right)} \right) \, = {\text{ f }}\left( {{\text{soil substrate}},{\text{ soil occupation}},{\text{ dynamics}},{\text{ forest stands}},{\text{ survey}}} \right)$$

We considered three different designs based on three different subsets of the overall data. These designs are the only complete factorial experiments that could be extracted from the overall experimental design for tick collection in Fig. 1. The subsets associated with designs 1, 2 and 3 were chosen to maintain the factorial structure and symmetry of the experiments, so as to preserve the predictive and explanatory capacity of the fitted models.

*Design 1*: a 2 × 4 × 4 factorial experiment with two replicates.

Factors:

- soil substrate (2 levels: CL, SA)
- soil occupation (4 levels: F, O, Me, W)
- survey (4 levels: S1, S2, S3, S4).

*Design 2*: a 2 × 2 × 4 factorial experiment with two replicates, within wetlands.

Factors:

- soil substrate (2 levels: CL, SA)
- dynamics (2 levels: C, Mis)
- survey (4 levels: S1, S2, S3, S4).

*Design 3*: a 2 × 2 × 3 × 4 factorial experiment with two replicates, within forests.

Factors:

- soil substrate (2 levels: CL, SA)
- dynamics (2 levels: R and S)
- forest stands (3 levels: Co, D, Mix)
- survey (4 levels: S1, S2, S3, S4).

For each design, we determined the best-fit model using a stepwise procedure with forward selection and backward elimination of variables. This procedure was achieved by minimizing the Akaike information criterion (AIC) while ensuring that the main effects and associated two-way interactions were statistically significant. We used a significance threshold set at α = 0.01 to overcome type I error inflation for the statistical tests.

Statistical analyses were performed using R software (version 4.2.1). PCA and CA were carried out with the “FactoMineR” package, while negative binomial regression required the use of the “MASS” package.

### Machine learning analysis

To determine the overall effects of different soil and habitat features on the abundance of ticks and pathogens, as well as the relative importance of individual predictors, we used several machine learning algorithms implemented in the python sklearn libraries (software Python 3.7 with Spyder (Ananconda3)). Unlike the analysis carried out with the GLM model described earlier in this paper, we also used machine learning models to be able to simultaneously analyze the impact of all the features (more than thirty features) on the abundance of ticks. We first selected features by removing one of each pair of highly correlated predictors (correlation > 90%). We then built a pipeline, consisting of random forest, adaboosting regressor, gradient boosting, xgboosting, lasso regressor, KNN, and support vector machine algorithms. We use a five-fold cross-validation process by randomly splitting data into k = 5 folds. The models are trained on the k − 1 = 4 folds, while one-fold is left to test a model. In this paper, all data is split into training and testing datasets, and a training dataset is used for cross-validation. We used R-Squared (R2), Mean Squared Error (MSE) and Root Mean Squared Error (RMSE) as performance measures to assess estimation performance of each model. For each of our seven models, several parameters have been integrated to optimize their performance. With our optimal model, the pipeline then reports the relative importance of the different input variables on the observed abundance of ticks or pathogens. We additionally evaluated the final model using Shap^70^ (SHapley Additive exPlanations), which provides a more thorough evaluation of the relationships between the inputs and the outputs of a model. Specifically, Shap quantifies and visualizes the positive or negative contributions of each of the input variables on the output variable being considered.

## Supplementary Information

Supplementary Information.

## Data Availability

Raw data are available at https://bul.univ-lorraine.fr/index.php/s/FzNyG6NiMBRoKtD.
