# Supplementary material for: Multiple factors affecting Ixodes ricinus ticks and associated pathogens in European temperate ecosystems (northeastern France)
Source: Sci Rep. 2024 Apr 24;14:9391. doi: 10.1038/s41598-024-59867-x (PMC11579317; doi:10.1038/s41598-024-59867-x)
Supplement: Supplementary file 1 — Supplementary Information. [file 41598_2024_59867_MOESM1_ESM.docx]

**Supplementary Material**

**Supplementary Material 1**. Soil characteristics at each study site.

| **Code** | **Parcel number** | **Bulk density (g/cm^3^)** | **CaCO_3_**  **(%)** | **Organic carbon**  **(%)** | **C:N** | **CEC**  **(cmol+/kg)** | **pH** | **Clay**  **0 - 2µm (%)** | **Silt**  **2 - 50 µm (%)** | **Sand**  **50 - 2000 µm (%)** |
| --- | --- | --- | --- | --- | --- | --- | --- | --- | --- | --- |
| CL-O | 1 | 0.9 | 4.8 | 4.314 | 10.8 | 31.2 | 7.64 | 53.9 | 40.6 | 5.5 |
|  | 2 | 0.94 | 5.9 | 5.482 | 11.2 | 35.7 | 7.62 | 63.8 | 33.5 | 2.7 |
| CL-Me | 3 | 1.1 | 0 | 3.17 | 11.3 | 18.3 | 6.25 | 31 | 57.3 | 11.7 |
|  | 4 | 1.16 | 4.7 | 5.246 | 9,0 | 36.2 | 7.51 | 64.1 | 32.6 | 3.3 |
| CL-W-C | 5 | 0.91 | 6.1 | 3.748 | 10.4 | 27.2 | 7.68 | 36 | 50.8 | 13.2 |
|  | 6 | 1.2 | 0 | 1.61 | 10.7 | 14.4 | 5.35 | 30.2 | 58 | 11.8 |
| CL-W-Mis | 7 | 0.94 | 0 | 4.79 | 12.6 | 25.7 | 5.79 | 48 | 49.8 | 2.2 |
|  | 8 | 1.13 | 0 | 4.29 | 11.3 | 26.8 | 6.48 | 39 | 51.9 | 9.1 |
| CL-F-R-Co | 9 | 0.94 | 0 | 4.67 | 12.6 | 33.6 | 7.54 | 50.5 | 44.5 | 5 |
|  | 10 | 0.98 | 0 | 3.38 | 11.7 | 20.1 | 5.12 | 41.2 | 51.5 | 7.3 |
| CL-F-S-Co | 11 | 1.23 | 0 | 5.42 | 15.1 | 13.2 | 4.83 | 26.6 | 65.7 | 7.7 |
|  | 12 | 0.91 | 0 | 3.17 | 12.7 | 10.9 | 4.52 | 30.8 | 60.6 | 8.6 |
| CL-F-R-D | 13 | 0.91 | 7.5 | 8.44 | 13.6 | 45.4 | 7.46 | 71.1 | 27.7 | 1.2 |
|  | 14 | 1,00 | 0 | 6.45 | 13.2 | 38.3 | 6.82 | 57.3 | 40.3 | 2.4 |
| CL-F-S-D | 15 | 0.9 | 0 | 6.67 | 12.1 | 37.9 | 6.19 | 61.7 | 34.7 | 3.6 |
|  | 16 | 1.15 | 0 | 2.46 | 15.4 | 5.5 | 4.41 | 21.3 | 68.1 | 10.6 |
| CL-F-R-Mix | 17 | 0.99 | 0 | 3.35 | 12,0 | 12.7 | 4.93 | 26.9 | 67 | 6.1 |
|  | 18 | 0.8 | 1.5 | 5.56 | 11.8 | 38,0 | 7.33 | 56.1 | 40.8 | 3.1 |
| CL-F-S-Mix | 19 | 1.06 | 0.8 | 3.574 | 10.5 | 35.5 | 7.32 | 54.2 | 42.6 | 3.2 |
|  | 20 | 0.97 | 0 | 2.22 | 13.1 | 6.9 | 4.45 | 20.8 | 68.9 | 10.3 |
| SA-O | 21 | 1.09 | 0 | 2.95 | 12.3 | 5,0 | 5.89 | 6.6 | 8.6 | 84.8 |
|  | 22 | 1.18 | 0 | 1.48 | 10.6 | 3.2 | 6.26 | 4.4 | 5 | 90.6 |
| SA-Me | 23 | 1.45 | 0 | 2.81 | 13.4 | 1.7 | 5.9 | 5.6 | 7.5 | 86.9 |
|  | 24 | 0.88 | 0 | 5.22 | 14.1 | 3.4 | 5.56 | 6.9 | 10.5 | 82.6 |
| SA-W-C | 25 | 0.7 | 0 | 3.63 | 14,0 | 1.8 | 4.67 | 4.9 | 3.3 | 91.8 |
|  | 26 | 1.14 | 0 | 4.4 | 12.9 | 3.5 | 5.28 | 5.6 | 7.6 | 86.8 |
| SA-W-Mis | 27 | 0.89 | 0 | 5.15 | 12.9 | 14.2 | 6.52 | 7.9 | 6.5 | 85.6 |
|  | 28 | 0.91 | 0 | 7.28 | 14.6 | 4.2 | 4.97 | 10.6 | 8.5 | 80.9 |
| SA-F-R-Co | 29 | 1.02 | 0 | 5.35 | 23.3 | 3,0 | 3.85 | 4.4 | 6.5 | 89.1 |
|  | 30 | 1.22 | 0 | 5.49 | 15.7 | 2.1 | 4,00 | 4.5 | 3.4 | 92.1 |
| SA-F-S-Co | 31 | 1.19 | 0 | 5.74 | 17.9 | 5.1 | 3.76 | 6.4 | 5.5 | 88.1 |
|  | 32 | 1.16 | 0 | 4.79 | 20,0 | 4.1 | 3.76 | 5.1 | 5.1 | 89.8 |
| SA-F-R-D | 33 | 1.01 | 0 | 7.47 | 17,0 | 8,0 | 5.15 | 9.4 | 8.9 | 81.7 |
|  | 34 | 1.25 | 0 | 3.13 | 14.2 | 1.8 | 4.49 | 6.4 | 7.9 | 85.7 |
| SA-F-S-D | 35 | 1.12 | 0 | 8.29 | 19.3 | 3.9 | 3.84 | 7.4 | 7.6 | 85 |
|  | 36 | 1.21 | 0 | 4.14 | 18.8 | 2.8 | 4.02 | 4.8 | 6.1 | 89.1 |
| SA-F-R-Mix | 37 | 1.24 | 0 | 2.91 | 15.3 | 2.7 | 4.63 | 7.2 | 15.8 | 77 |
|  | 38 | 0.95 | 0 | 5.59 | 21.5 | 4,0 | 3.87 | 7.6 | 7.6 | 84.8 |
| SA-F-S-Mix | 39 | 1.07 | 0 | 4.8 | 20.9 | 4.9 | 3.82 | 5.2 | 8 | 86.8 |
|  | 40 | 1.06 | 0 | 6.59 | 20,0 | 4.2 | 3.95 | 5.2 | 6.9 | 87.9 |

**Supplementary Material 2.** Confidence intervals for regression coefficients presented in Table 3.

| **Negative binomial regression** | | | | |
| --- | --- | --- | --- | --- |
| **Equation** | **Variable** | **Regression coefficient** | **Standard error** | **95% confidence interval** |
| 3  Nymphs | Intercept  CL  Me  O  S2  S3  CL*Me  CL*O  CL*W | 0.7797  1.0892  -0.5034  -0.8404  0.2517  0.2477  -1.3724  -1.3817  -1.5239 | 0.0812  0.0786  0.1727  0.2020  0.0910  0.0910  0.2604  0.3168  0.1610 | [0.6205; 0.9389]  [0.9351; 1.2433]  [-0.8419; -0.1649]  [-1.2363; -0.4445]  [0.0733; 0.4301]  [0.0693; 0.4261]  [-1.8828; -0.8620]  [-2.0026; -0.7608]  [-1.8395; -1.2083] |
| 4  Nymphs | Intercept  CL  Mis | 1.0160  0.4258  -0.4650 | 0.1537  0.1788  0.1788 | [0.7147; 1.3173]  [0.0754; 0.7762]  [-0.8154; -0.1146] |
| 5  Nymphs | Intercept  CL  D  Mix  S3  S4  S*S2  S*S3  CL*S2  CL*S3  CL*S4 | 1.0797  0.7337  -0.2193  -0.1733  0.2966  -0.3184  -0.3840  -0.5157  0.5414  0.3241  0.4966 | 0.1062  0.1107  0.0678  0.0675  0.1375  0.1481  0.1108  0.1094  0.1577  0.1561  0.1623 | [0.8715; 1.2879]  [0.5167; 0.9507]  [-0.3522; -0.0864]  [-0.3056; -0.0410]  [0.0271; 0.5661]  [-0.6087; -0.0281]  [-0.6012; -0.1668]  [-0.7301; -0.3013]  [0.2323; 0.8505]  [0.0181: 0.6301]  [0.1785; 0.8147] |
| 6  Infected | CL  O  S3  CL*Me  CL*W | 1.1987  -2.1758  0.6723  -1.4514  -0.6150 | 0.2063  0.5976  0.2053  0.6041  0.2028 | [0.7944; 1.6030]  [-3.3471; -1.0045]  [0.2699; 1.0747]  [-2.6354; -0.2674]  [-1.0125; -0.2175] |
| 7  Infected | CL  Mis  S2  S3 | 0.5009  -0.5311  0.6046  0.7016 | 0.1748  0.1755  0.2657  0.2627 | [0.1583; 0.8435]  [-0.8751; -0.1871]  [0.0838; 1.1254]  [0.1867; 1.2165] |
| 8  Infected | CL  S3  S*D  S*S2  S*S3 | 1.0952  0.6896  -0.4730  -0.7056  -0.5522 | 0.1934  0.2265  0.1836  0.2212  0.2109 | [0.7161; 1.4743]  [0.2457; 1.1335]  [-0.8329; -0.1131]  [-1.1392; -0.2720]  [-0.9656; -0.1388] |
| **Logistic regression** | | | | |
| **Equation** | **Variable** | **Regression coefficient** | **Odds ratio** | **95% confidence interval** |
| 9  BOR | Intercept  W  S3 | -2.3247  0.5719  0.4356 | 0.0980  1.7717  1.5458 | [0.0753; 0.1249]  [1.3311; 2.3347]  [1.1272; 2.1345] |
| 10  HTRF | Intercept  S4 | -3.3730  -1.0604 | 0.0343  0.3463 | [0.0222; 0.0503]  [0.1514; 0.7247] |
| 11  ANA | Intercept  S4 | -3.6138  1.0724 | 0.0269  2.9225 | [0.0165; 0.0412]  [1.7511; 5.0925] |
| 12  TOTAL | Intercept  W  S3  S4 | -1.7413  0.3968  0.3139  0.3281 | 0.1753  1.4871  1.3688  1.3883 | [0.1429; 0.2150]  [1.1555; 1.9137]  [1.0497; 1.7848]  [1.0610; 1.8167] |

Abbreviations: BOR, *Borrelia burgdorferi* sensu lato; HTRF, *Borrelia miyamotoi*; ANA, *Anaplasma phagocytophilum;* CL, Clay-Limestone; Me, Meadow; O, Orchard; W, Wetland; Mis, Missing; S, Stable; D, Deciduous; S1, S2, S3, S4, the four surveys.

**Supplementary Material 3. Correlation analysis.**

We performed a correlation analysis among nymph abundance, pathogen abundance, camera trap-based indicators of faunal presence (the contact index [CI] and diversity indicator [DI]), and two soil characteristics (soil moisture and respiration). The most relevant statistically significant associations are presented below.

Nymph abundance was positively correlated with CI (τ=0.2287; p<0.01), DI (τ=0.2449; p<0.01), and soil moisture (τ =0.2529; p<10^-4^).

ANA abundance was positively correlated with CI (τ=0.1750; p=0.0219), DI (τ=0.2122; p<0.01), soil moisture (τ =0.1747; p=0.0212) and respiration (τ=0.2590; p<0.01). The relative abundance of ANA (expressed relative to the abundance of all pathogens) was positively correlated with DI (τ=0.1987; p=0.0122) and soil respiration (τ=0.2785; p<0.01).

Finally, both soil moisture and the overall abundance of infected nymphs were positively correlated with soil respiration (τ=0.1744; p<0.01 and τ=0.1563; p=0.0267, respectively), indicating that soil moisture conditions were favorable for both microbial soil respiration and *I. ricinus* nymph survival.

**Supplementary Material 4**. Model performance ranking for machine learning models predicting abundance of nymphs [AM1]. Abbreviations: RMSE, root-mean squared error.

| Rank of Models | Mean fit  time | Mean score  time | RMSE | R^2^ Std of cross  validation | R^2^ of cross  validation | R^2^ of  test |
| --- | --- | --- | --- | --- | --- | --- |
| Xgboost  regressor | 2.83642 | 0.108311 | 1.287611 | 0.128441 | 0.585995 | 0.662636 |
| AdaBoost  regressor | 2.14187 | 0.0063827 | 1.298696 | 0.0713132 | 0.609151 | 0.656803 |
| Gradient boosting regressor | 0.367219 | 0.00219393 | 1.300325 | 0.0688651 | 0.662176 | 0.655941 |
| Support Vector Regression | 0.000996923 | 0.000398874 | 1.374108 | 0.0960419 | 0.608487 | 0.615788 |
| KNN Regression | 0.00119691 | 0.00139618 | 1.418612 | 0.27642 | 0.410072 | 0.590498 |
| Random Forest Regressor | 0.267883 | 0.0107714 | 1.479983 | 0.0827546 | 0.551773 | 0.554300 |
| Lasso Regression | 0.00119638 | 0.000799227 | 1.630691 | 0.0749851 | 0.655477 | 0.458906 |

**Supplementary Material 5**. Model performance ranking for machine learning models predicting the abundance of the ANA pathogen [AM2].

| Rank of Models | Mean fit  time | Mean score  time | RMSE | R^2^ Std of cross  validation | R^2^ of cross  Validation | R^2^ of  test |
| --- | --- | --- | --- | --- | --- | --- |
| Xgboost  regressor | 0.00119662 | 0.00719767 | 0.673621 | 0.0714269 | 0.388001 | 0.262054 |
| KNN Regression | 0.144814 | 0.00418797 | 0.686957 | 0.231263 | 0.254654 | 0.232546 |
| Random Forest Regressor | 0.212232 | 0.0159587 | 0.700422 | 0.108573 | 0.402778 | 0.202166 |
| AdaBoost  regressor | 3.52936 | 0.137232 | 0.702981 | 0.0961297 | 0.489616 | 0.196326 |
| Lasso Regression | 0.00339222 | 0.00119605 | 0.726281 | 0.196788 | 0.34721 | 0.142169 |
| Gradient boosting regressor | 0.660034 | 0.00299196 | 0.750736 | 0.11122 | 0.571837 | 0.083427 |
| Support Vector Regression | 0.00100689 | 0.000791883 | 0.756758 | 0.16514 | 0.419101 | 0.068662 |

**Supplementary Material 6**. Model performance ranking for machine-learning models predicting the abundance of the BOR pathogen [AM3].

| Rank of Models | Mean fit  time | Mean score  time | RMSE | R^2^ Std of cross  validation | R^2^ of cross-  validation | R^2^ of  test |
| --- | --- | --- | --- | --- | --- | --- |
| Xgboost  regressor | 2.46661 | 0.115692 | 0.907117 | 0.100758 | 0.406801 | 0.542695 |
| AdaBoost  regressor | 2.34672 | 0.00518746 | 0.911862 | 0.132113 | 0.370305 | 0.537898 |
| Random Forest Regressor | 0.267485 | 0.0129653 | 0.933936 | 0.105832 | 0.407119 | 0.515255 |
| Gradient boosting regressor | 0.13484 | 0.00199475 | 0.951088 | 0.110034 | 0.423987 | 0.497287 |
| Lasso Regression | 0.00159602 | 0.000991583 | 1.011785 | 0.118211 | 0.467539 | 0.431073 |
| KNN Regression | 0.00120912 | 0.00159278 | 1.097002 | 0.136333 | 0.340323 | 0.331203 |
| Support Vector Regression | 0.000993013 | 0.000602961 | 1.121473 | 0.110523 | 0.316469 | 0.301032 |

**Supplementary Material 7**. Model performance ranking for machine-learning models predicting the abundance of the HTRF pathogen [AM4].

| Rank of Models | Mean fit  time | Mean score  time | RMSE | R^2^ Std of cross  validation | R^2^ of cross  validation | R^2^ of  test |
| --- | --- | --- | --- | --- | --- | --- |
| Xgboost  regressor | 0.000637579 | 0.000996065 | 0.455338 | 0.0895676 | 0.122499 | 0.312998 |
| Lasso Regression | 0.35009 | 0.00438833 | 0.523203 | 0.201798 | 0.0385235 | 0.092953 |
| Gradient boosting regressor | 0.0875015 | 0.00119739 | 0.529735 | 0.062408 | 0.155222 | 0.070164 |
| Random Forest Regressor | 0.217545 | 0.0101838 | 0.538362 | 0.134625 | 0.153122 | 0.039632 |
| AdaBoost  regressor | 0.962027 | 0.050066 | 0.545638 | 0.129266 | 0.0661071 | 0.013496 |
| KNN Regression | 0.00188823 | 0.0022161 | 0.572304 | 0.105775 | -0.0337426 | -0.085283 |
| Support Vector Regression | 0.00102863 | 0.000205326 | 0.575980 | 0.126246 | 0.123484 | -0.099270 |

**
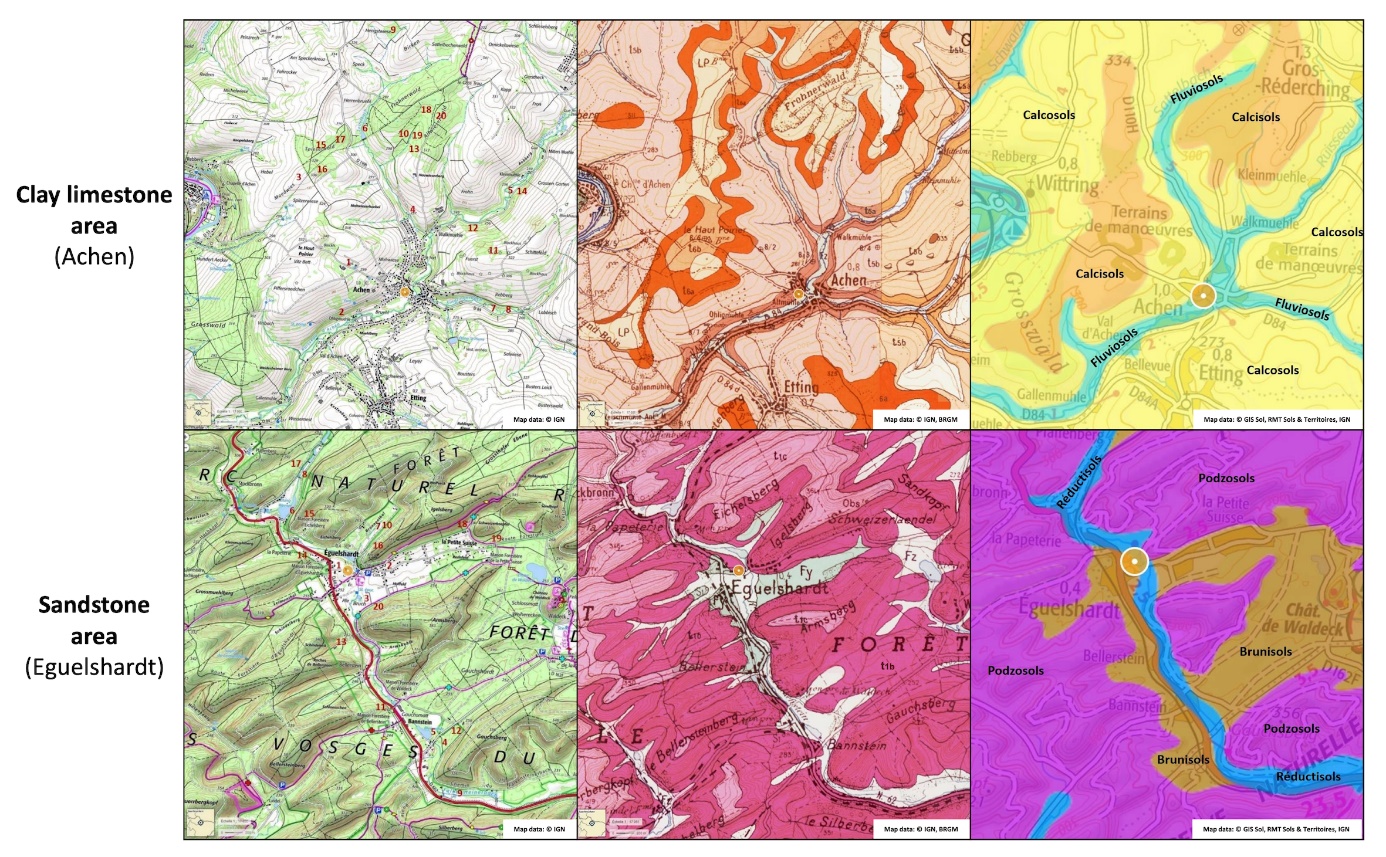
**

**Supplementary Material 8**. Topographic 1:25,000 maps (*left*), geologic maps (*center*), and pedologic maps (*right*) of selected study sites, separated by soil substrate. Geological map legend: LP, Silty covers; Fz, Recent alluvial deposits; Fy, Ancient alluvial deposits; t6b, Variegated clays; t6a, Lower dolomite; t5b, Ceratites layers; t5a, Crinoidal limestone; t1c, Upper Vosges sandstone; t1b, Lower Vosges sandstone.

**Supplementary Material 9**. Characteristics of the 40 tick sampling sites.

| **Code** | **Parcel number** | **Latitude** | **Longitude** | **Elevation (m)** | **Slope (%)** | **Humus forms** |
| --- | --- | --- | --- | --- | --- | --- |
| CL-O | 1 | 49° 2' 47,9'' | 7° 10' 24,6'' | 306 | 8 | Carbonated grassland Eumull |
|  | 2 | 49° 2' 27,5'' | 7° 10' 15,3'' | 260 | 25 | Carbonated grassland Eumull |
| CL-Me | 3 | 49° 3' 23,6'' | 7° 9' 55,8'' | 339 | 2 | Grassland Eumull |
|  | 4 | 49° 3' 8,2'' | 7° 11' 0,7'' | 259 | 16 | Carbonated grassland Eumull |
| CL-W-C | 5 | 49° 3' 18,4'' | 7° 12' 1,2'' | 254 | 2 | Carbonated grassland Eumull |
|  | 6 | 49° 3' 41,7'' | 7° 10' 30,7'' | 294 | 5 | Grassland Eumull |
| CL-W-Mis | 7 | 49° 2' 28,4'' | 7° 11' 52,9'' | 268 | 4 | Grassland Eumull |
|  | 8 | 49° 2' 28,7'' | 7° 12' 1,1'' | 266 | 8 | Grassland Eumull |
| CL-F-R-Co | 9 | 49° 4' 20,4'' | 7° 10' 49,3'' | 314 | 8 | Oligomull |
|  | 10 | 49° 3' 37,9'' | 7° 10' 58,1'' | 295 | 8 | Mesomull |
| CL-F-S-Co | 11 | 49° 3' 36,8'' | 7° 10' 6,3'' | 329 | 8 | Oligomull |
|  | 12 | 49° 3' 24,9'' | 7° 10' 5,9'' | 323 | 10 | Dysmull |
| CL-F-R-D | 13 | 49° 2' 52,9'' | 7° 11' 53,7'' | 313 | 11 | Carbonated Eumull |
|  | 14 | 49° 3' 2,8'' | 7° 11' 38'' | 262 | 23 | Eumull |
| CL-F-S-D | 15 | 49° 3' 41,1'' | 7° 10' 18,8'' | 316 | 8 | Mesomull |
|  | 16 | 49° 3' 45,5'' | 7° 11' 12,1'' | 328 | 5 | Dysmull |
| CL-F-R-Mix | 17 | 49° 3' 35,2'' | 7° 11' 1,4'' | 305 | 6 | Oligomull |
|  | 18 | 49° 3' 18,5'' | 7° 12' 8,9'' | 265 | 15 | Carbonated Eumull |
| CL-F-S-Mix | 19 | 49° 3' 38,8'' | 7° 11' 1,5'' | 305 | 6 | Carbonated Eumull |
|  | 20 | 49° 3' 45,7'' | 7° 11' 16,4'' | 323 | 8 | Dysmull |
| SA-O | 21 | 49° 1' 17,6'' | 7° 29' 32'' | 257 | 2 | Grassland Moder |
|  | 22 | 49° 1' 17,9'' | 7° 30' 5,4'' | 269 | 9 | Grassland Moder |
| SA-Me | 23 | 49° 1' 3,3'' | 7° 29' 50,5'' | 254 | 3 | Grassland Moder |
|  | 24 | 49° 0' 12,8'' | 7° 30' 31,5'' | 234 | 4 | Grassland Moder |
| SA-W-C | 25 | 49° 0' 14,8'' | 7° 30' 25,2'' | 237 | 1 | Grassland Moder |
|  | 26 | 49° 1' 35,4'' | 7° 29' 2,6'' | 266 | 7 | Grassland Moder |
| SA-W-Mis | 27 | 49° 1' 32,5'' | 7° 29' 57'' | 259 | 8 | Grassland Moder |
|  | 28 | 49° 1' 51'' | 7° 29' 11,5'' | 260 | 6 | Grassland Hydromoder |
| SA-F-R-Co | 29 | 48° 59' 53,4'' | 7° 30' 40'' | 242 | 14 | Dysmoder |
|  | 30 | 49° 1' 32,7'' | 7° 29' 59'' | 259 | 8 | Eumoder |
| SA-F-S-Co | 31 | 49° 1' 35'' | 7° 29' 17,1'' | 330 | 29 | Eumoder |
|  | 32 | 49° 1' 23,8'' | 7° 29' 52,8'' | 286 | 45 | Mor |
| SA-F-R-D | 33 | 49° 0' 26,2'' | 7° 29' 56,2'' | 251 | 11 | Hemimoder |
|  | 34 | 49° 0' 15,7'' | 7° 30' 37,4'' | 245 | 7 | Hemimoder |
| SA-F-S-D | 35 | 49° 1' 59'' | 7° 29' 13,9'' | 278 | 8 | Dysmoder |
|  | 36 | 49° 1' 32,2'' | 7° 30' 47'' | 285 | 27 | Eumoder |
| SA-F-R-Mix | 37 | 49° 0' 50,1'' | 7° 29' 38,8'' | 252 | 26 | Eumoder |
|  | 38 | 49° 1' 21,2'' | 7° 29' 15'' | 265 | 10 | Dysmoder |
| SA-F-S-Mix | 39 | 49° 1' 26,1'' | 7° 30' 56,2'' | 279 | 15 | Dysmoder |
|  | 40 | 49° 1' 1,7'' | 7° 29' 52,5'' | 265 | 42 | Eumoder |

**Supplementary Material 10**. Floristic inventory of studied plots

| **Code** | **Parcel number** | **Floristic inventory** |
| --- | --- | --- |
| CL-O | 1 & 2 | **Orchards** |
| CL-Me | 3 & 4 | **Meadow (mesophilic and eutrophic), dominated by high herbs**  **3:** *Arrhenatherion elatioris* alliance: *Arrhenatherum elatius, Dactylis glomerata, Festuca rubra*. Other species: *Trifolius pratense, Achillea millefolium, Galium mollugo, Plantago lanceolata, Lotus corniculatus, Calystegia sepium, Avenula pratensis, Allium vineale.*  **4:** *Arrhenatherion elatioris* alliance: *Arrhenatherum elatius, Dactylis glomerata, Festuca rubra)*. Other species: *Achillea millefolium, Galium verum, Ononis spinosa, Lotus corniculatus, Rumex acetosa, Ranunculus acris, Scabiosa columbaria, Allium vineale.* |
| CL-W-C | 5 & 6 | **5: Edge forest/meadow (meso-hygrophilic)**  Alluvial forest : Alno-Padion alliance (*Fraxinus excelsior, Alnus glutinosa, Salix* sp*, Acer pseudoplatanus, Acer campestre*).  Meadow: *Arrhenaterion elatioris* alliance: *Arrhenatherum elatius, Dactylis glomerata, Festuca rubra*.  The meadow also includes other species according to the microtopography of the area. Some of these species are nitrophilic and include the ruderal species (Galio Urticetea class) *Urtica dioica, Galium aparine, Convolvulus sepium,* *Cirsium arvense,* and *Equisetum arvense.* Others are mesophilic (Arrhenatalia order): *Arrhenaterum elatius, Achillea millefolium, Dactylis glomerata, Avenula pratensis, Holcus lanatus*). Others include hygrophilic species in the Alno-Padion alliance: *Geum urbanum, Alliaria officinalis, Glechoma hederacea.*  **6: Meadow (meso-hygrophilic) and adjacent small woods along the pond**  This meadow includes high herbs (1-3 m) in the Galio-Urticetea class (*Urtica dioica, Galium aparine, Calystegia sepium, Equisetum arvense*), mesophilic meadow species (*Arrhenaterum elatius, Achillea millefolium, Dactylis glomerata, Avenula pratensis, Holcus lanatus, Festuca rubra*), and hygrophilic forest species in the Alno-Padion and Caricion groups (*Carex* sp*, Geum urbanum, Alliaria officinalis, Fragaria vesca, Stachys sylvatica, Rubus fructicosus*)*.*  There is a small marsh and fallows area close to the pond.  Species in the small pond: *Salix* bushes, *Carex* (sp), Scirpus sp, *Phragmites communis, Phalaris arundinacea,* *Iris pseudacorus, Equisetum palustre, Lycopus europaeus, Scutellaria galericulata.*  Species of the fallow: *Galium mollugo, Geum urbanum, Eupatorium cannabinum, Erigeron* sp*, Hypericum* sp*, Arrhenaterum elatius, Festuca rubra, Potentilla anserina*, *Artemisia vulgaris, Alliaria officinalis, Galium aparine* Quelques rudérales: *Urtica dioica, Equisetum arvense, Artemisia vulgaris, Convolvulus polygonatum.*  The small woods of the area include *Picea excelsa* and *Hedera helix* as well as small trees and bushes typical of oak-hornbeam forest (Carpinion alliance): *Quercus robur, Carpinus betulus, Prunus avium*, *Rosa arvensis, Crataegus monogyna, Prunus spinosa*, *Deschampsia caespitosa, Hedera helix, Rubus fructicosus, Vicia sepium, Taraxacum dens leonis, Fragaria vesca, Glechoma hederacea, Alliaria officinalis, Brachypodium sylvaticum, Stachys sylvatica, Geum urbanum.* |
| CL-W-Mis | 7 & 8 | **7: Meadow (mesophilic and eutrophic), dominated by high herbs**  Arrhenatherion elatioris alliance: *Arrhenatherum elatius, Dactylis glomerata, Festuca rubra.*  Other species: *Trifolius pratense, Achillea millefolium, Galium album, Plantago lanceolata, Lotus corniculatus, Calystegia sepium, Avenula pratensis, Allium vineale.*  **8: Meadow (Meso-hygrophilic)**  Old channel of the Singlingerbach river colonized by a mesohygrophilic meadow with high herbs (Arrhenaterion elatioris alliance): *Arrhenaterum elatioris, Dactilis glomerata, Festuca rubra, Galium album, Sanguisorba major, Potentilla anserina, Filipendula ulmaria, Holcus lanatus, Viola* sp. |
| CL-F-R-Co | 9 & 10 | **9 and 10: Picea plantation**  Trees are planted in close proximity. The forest includes a few deciduous tree species (*Fagus sylvatica, Prunus avium, Betula pendula*)*.* |
| CL-F-S-Co | 11 & 12 | **11 and 12: Picea plantation**  These plantations are very dark; they have no substantial understory and a lot of dead wood. |
| CL-F-R-D | 13 & 14 | **13: Bush community**  This bush community (Prunetalia order) is composed of pioneer species: *Prunus spinosa, Corylus avellana, Clematis vitalba*, and a few trees (e.g., *Prunus avium*).  The regeneration area is species-rich and includes *Ligustrum vulgare, Carpinus betulus, Quercus robur, Cornus sanguinea, Acer campestre, Prunus spinosa, Prunus avium.*  Herbaceous species: *Brachypodium sylvaticum, Hedera helix, Anthriscus sylvestris, Geranium robertianum, Fragaria vesca, Geum urbanum.*  **14: Early-successional deciduous forest**  Colonizing trees: *Acer pseudoplatanus, Fraxinus excelsior*  Bushes: *Prunus spinosa, Crataegus monogyna* (Prunetalia order).  Herbaceous species are abundant, including nitrophilic species (*Urtica dioica*) and species from oak-hornbeam community: *Lamium galeobdolon, Stachys sylvatica, Lamium maculatum, Glechoma hederacea, Rubus caesius, Circaea lutetiana, Vicia sepium, Brachypodium pinnatum, Alliaria officinalis, Geranium robertianum, Filipendula ulmaria.* |
| CL-F-S-D | 15 & 16 | **15: Ancient beech forest: mesophilic**  Beech forest (Fagion alliance, Luzulo-Fagetum association): *Fagus sylvatica* (dominant), *Acer campestre, Prunus avium, Hedera helix, Quercus* sp*, Fraxinus excelsior*.  Important herbaceous coverage (50-80%): *Anemone nemorosa, Carex remota, Carex sylvatica, Hedera helix, Alliaria officinalis, Sanicula europaea, Geranium robertianum, Stachys sylvatica, Circaea lutetiana, Viola reichenbachiana, Vicia sepium, Glechoma hederacea, Campanula trachelium, Milium effusum*.  **16: Ancient beech forest**  Beech forest (Fagion alliance, Luzulo-Fagetum association) with a close canopy and many large woods. The beech is totally dominant. There are a few seedlings of other species (*Acer pseudoplatanus, Quercus sp, Prunus avium*). |
| CL-F-R-Mix | 17 & 18 | **17: Gir and oak plantation**  Plantation understory includes saplings of *Acer pseudoplatanus, Crataegus monogyna,* and *Prunus spinosa,* as well as the herbaceous species *Dactilis glomerata, Carex remota, Carex sylvatica, Rumex sanguineus,* and *Viola reichenbachiana*.  The edges of the plantation are species rich and include a liana (*Lonicera periclymenum*) and several herb species: *Geranium robertianum, Festuca gigantea, Glechoma hederacea, Campanula, Lychnis flos cocoli, Scrofularia nodosa, Angelica sylvestris,* *Urtica dioica, Juncus conglomeratus, Poa trivialis, Equisetum arvense, Eupatorium cannabinum.*  **18: Picea plantation**  This plantation has been abandoned and includes species from the oak-hornbeam community (Carpinion alliance): seedlings of *Acer campestre, A. pseudoplatanus, Fraxinus excelsior, Juglans regia, Quercus robur, Carpinus betulus, Cornus sanguinea, Crataegus monogyna, Evonymus europaeus, Corylus avellana, Clematis vitalba,*  Herbaceous species (about 20%): *Geranium robertianum, Hedera helix, Galium aparine, Dactilis glomerata, Milium effusum, Alliaria officinalis, Brachypodium sylvaticum, Rubus fructicosus, Viola sp*.  Some species are ruderal (*Urtica dioica, Bryonia dioica*) or issued from adjacent mesophilic meadows: *Holcus lanatus, Dactylis glomerata.* |
| CL-F-S-Mix | 19 & 20 | **19: Mixed plantation of Picea and deciduous species (*Quercus rubra, Fagus sylvatica*)**  These trees are planted separately at the same site. In the beech wood, the herbaceous coverage is only 5%: *Circaea lutetiana, Alliaria officinalis, Geranium robertianum*.  **20: Ancient beech forest**  Beech forest (Fagion alliance, Luzulo-Fagetum association) with a few regeneration species: *Fraxinus excelsior, Acer pseudoplatanus.* |
| SA-O | 21 & 22 | **Orchards** |
| SA-Me | 23 & 24 | **23: Meadow (Meso-hygrophilic)**  Arrhenatherion alliance.  **24: Meadow (Meso-hygrophilic) with a few mesophilic plants**  Protected area on sandy soils. Arrhenatherion alliance: *Arrhenaterum elatius* dominant*; Festuca rubra, Briza media, Galium mollugo, G. verum, Deschampsia flexuosa, Plantago media, Viola lutea, Sanguisorba major.*  Mesophilic facies*: Bromus erectus, Dianthus carthusianorum, Lathyrus pratensis*, *Hieracium pilosella.*  A few pioneer species*: Euphorbia cyparissias, Rumex acetosella, Thymus* sp., semis *Prunus spinose.* |
| SA-W-C | 25 & 26 | **25: Around a pond**  This pond is surrounded partly by a meadow and partly by a hygrophilic wood. The wood includes *Alnus glutinosa*, *Pinus sylvestris*, *Betula pubescens,* and hybrid poplars, with understory species *Rhamnus frangula* and *Rubus fructicosus* and an invasive species, *Solidago* sp.  In the open area*, Carex* sp., *Scirpus* sp., and *Juncus* sp. dominate. The area behind the meadow is composed of *Festuca tenuifolia, Holcus mollis, Stellaria graminea, Plantago lanceolata, Achillea millefolium,* and *Ranunculus repens.*  There are two invasive species*: Solidago* sp. and bamboos.  **26: Hygrophilic wood close to a pond**  The wood (Alno-Padion alliance) includes *Alnus glutinosa, Prunus padus,* *Carex sp.,* and *Equisetum palustre*.  On the edges: bushes of *Crataegus monogyna* and *Prunus spinosa* as well as mesohygrohilic herbs (*Sanguisorba major, Deschampsia flexuosa, Lysimachia vulgaris*). |
| SA-W-Mis | 27 & 28 | **27: Fallow (Meso-hygrophilic)**  Fallow colonizing an old meadow (Arrhenatheron elatioris alliance). The colonizing species are *Sarothamnus scoparius* along with a few bushes of *Prunus spinosa.*  Grassland species: *Arrhenaterum elatius, Dactylis glomerata, Cynosorus cristatus, Deschampsia flexuosa, Agropyron repens*, and others (*Thymus sp, Euphorbia cyparissias, Galium album, Myosotis arvensis, Galium verum, Campanula sp., Achillea millefolium*)*.*  Marked presence of ruderal species: *Urtica dioica, Eupatorium cannabinum, Rumex acetosella, Rubus fructicosus.*  Dense presence of exotic species: *Solidago, Erigeron* sp*.*  **28: Meadow (Meso-hygrophilic) close to small ponds**  Meadow including species of *Arrhenaterion elatioris*: A*rrhenaterum elatius, Dactylis glomerata, Holcus lanatus, Anthoxanthum odoratum, Briza media, Alopecurus pratensis, Plantago lanceolata, Stellaria graminea, Campanula* sp. |
| SA-F-R-Co | 29 & 30 | **29: Picea species**  Plantation of 10 m width, between a forest path and a meadow. The plantation is bordered by a few deciduous species (*Fagus, Populus, Carpinus*).  **30: Picea plantation**  Narrow plantation, with a wild apple tree along the path. A few herbs are present in the understory (e.g., *Viola reichenbachiana*), along with oak seedlings. |
| SA-F-S-Co | 31 & 32 | **31: Exotic fir plantation**  Exotic fir has been planted in a half-open site, with a few beeches in understoreys. Soil bryophytes, ferns and a lot of dead wood.  **32: Fir and spruce forest**  Forest growing on a steep slope. The soil is covered with Bryophytes at 90%, and a few seedlings of *Fagus* and *Picea.* Dead wood on the soil  . |
| SA-F-R-D | 33 & 34 | **33: Spontaneous young wood**  The wood is close to an abandoned train track. The wood is rich in dead wood. It is composed of deciduous species *(Fagus sylvatica, Tilia, Carpinus betulus)* with a few species in the understorey (seedlings of oak, *Anemone nemorosa, Viola reichenbachiana*).  **34: Spontaneous young oak forest**  Young forest composed of *Quercus robur, Populus tremula.* In the understoreys, seedlings of one exotic species (*Laurus* sp), *Carpinus betulus, Picea excelsa, Populus tremula, Pinus sylvestris, Prunus avium, Rosa arvensis, Sarothamnus scoparius*. Herbaceous species: *Arrhenaterum elatius* dominant, *Holcus lanatus, Juncus* sp*, Stachys sylvatica, Carex sylvatica, Poa trivialis, Galium aparine, Crepis* sp*, Moeringia trinervia, Galeopsis tetrahit, Alliaria officinalis, Euphorbia cyparissias, Poygonum convolvulus.* |
| SA-F-S-D | 35 & 36 | **35: Ancient beech forest**  The forest is dominated by the beech with a few oaks. Beautiful forest architecture with close canopy and dead wood. Nearly no herbs.  **36: Old oak beech forest**  The understory is rich: a few hornbeam and beech.  A few seedlings of *Quercus robur, Carpinus betulus, Fagus sylvatica, Picea excelsa,* *Rhamnus frangula. Carex remota.*  Along the pathway, the soil coverage is 100%: *Dactilis glomerata, Rubus fructicosus, Euphorbia cyparissia, Agrostis stolonifera, Brachypodium sylvaticum, Teucrium sp, Alliaria officinalis, Hypericum perforatum,* horticultural plant (Iris). |
| SA-F-R-Mix | 37 & 38 | **37: Young beech forest**  Beech forest with a few poplars (*Populus tremula*) in gaps and edges, and a few dead spruces. Very few species in the soil (*Anemone nemorosa*).  **38: Mixed young forest**  Forest composed of young *Picea excelsa, Fagus sylvatica*, and a few birches (*Betula pendula*). |
| SA-F-S-Mix | 39 & 40 | **39: Beech and Pine wood**  This forest is situated at the bottom of a small valley. Canopy trees are beech, pine (*Pinus sylvestris)* and a few oaks (*Quercus* sp.).  On the soil: *Vaccinium myrtillus, Deschampsia flexuosa*, seedlings of *Sarothamnus scoparius.*  **40: Ancient beech forest and fallow (Mesophilic)**  Beech forest including several conifers (pine, fir, Douglas fir). Understory is scarce, with a few beeches.  A few herbs: *Poystichum spinusosum, Luzula maxima.*  Close to the forest, there is:   1. A mesophilic meadow (*Arrhenatherion elatioris*): *Achillea millefolium, Campanula trachelium, Plantago lanceolata, Dactilis glomerata, Trifolium pratense, Taraxacum dens leonis, Galium album, Bromus erectus, Rumex acetosella, Rumex acetosa, Thlaspi sp, Artemisia arvense, Alopecurus pratense, Vicia sepium.* 2. A fallow dominated by *Artemisia arvense.* |
